# Fishing with two lines: a hybrid approach to spatial transcriptomics discovery

**DOI:** 10.26508/lsa.202603690

**Published:** 2026-07-27

**Authors:** Arianna L Williams-Katek, Saahithi Mallapragada, Evan D Mee, Brandon K Fischer, Annika Vannan, Laurie C Eldredge, Gail H Deutsch, Jonathan A Kropski, Jennifer MS Sucre, Nicholas E Banovich

**Affiliations:** 1 Division of Bioinnovation and Genome Sciences, Translational Genomics Research Institute (TGen) , Phoenix, AZ, USA; 2 School of Life Sciences, Arizona State University , Tempe, AZ, USA; 3 Division of Pulmonary and Sleep Medicine, Department of Pediatrics, University of Washington School of Medicine , Seattle, WA, USA; 4 Seattle Children’s Hospital , Seattle, WA, USA; 5 Center for Respiratory Biology and Therapeutics, Seattle Children’s Research Institute , Seattle, WA, USA; 6 Department of Laboratory Medicine and Pathology, University of Washington and Seattle Children’s Hospital , Seattle, WA, USA; 7 Department of Cell and Developmental Biology, Vanderbilt University , Nashville, TN, USA; 8 Division of Allergy, Pulmonary and Critical Care Medicine, Department of Medicine, Vanderbilt University Medical Center , Nashville, TN, USA; 9 Department of Veterans Affairs Medical Center , Nashville, TN, USA; 10 Biodevelopmental Origins of Lung Disease (BOLD) Center, Vanderbilt University School of Medicine , Nashville, TN, USA; 11 Division of Neonatology, Department of Pediatrics, Vanderbilt University Medical Center , Nashville, TN, USA

## Abstract

Spatial transcriptomics faces a trade-off between the number of genes assayed and depth of per-gene sensitivity. We developed a “dual-chemistry” method that combines the high sensitivity of a 10x Genomics Xenium V1 custom panel (up to 480 genes) with the broad coverage of the Prime 5K panel (5,001 genes) on a single tissue section. This involved co-hybridizing Prime and V1 probes and sequentially running the V1 and Prime decoding chemistries. Applied to a human lung tissue microarray, we observed high concordance between the V1 and Prime chemistries when run independently (on serial sections) and the dual-chemistry runs. Overlapping genes (profiled on both V1 and Prime chemistries) showed similar expression patterns in the dual run demonstrating the fidelity of the assay. By combining information from both the V1 and Prime chemistries within the same cell, we retain more cells, gain valuable additional information, and enable both high-sensitivity profiling and discovery.

## Abstract (precis)

This study serves as a technical proof of concept demonstrating the hybridization and co-detection of both low- and high-plex probe chemistries on a single Xenium spatial transcriptomics slide with 17 hLung samples.

## Introduction

The advent of commercially available platforms for profiling of gene expression changes at cellular or subcellular resolution, collectively known as spatial transcriptomics, is revolutionizing how researchers study tissue biology. Current commercially available instruments are largely split into two types of assays: imaging-based and sequencing-based. Imaging-based technologies generally depend on sequential cycles of in situ hybridization to decode transcript identity based on patterns of binding across cycles (1, 2, 3). Sequencing-based technologies generally rely on a pull-down method of probe or transcript binding to a separate capture oligonucleotide (4). The transcripts can then undergo library preparation and sequencing, then be mapped back to a barcoded coordinate grid based on the original tissue position on the slide. Across all platforms, there is a trade-off between breadth and depth. In general, lower plex imaging–based platforms provide high per-gene sensitivity, whereas high-plex imaging and sequencing-based platforms allow for broader coverage (5, 6 *Preprint*). An idealized platform would deliver whole transcriptome coverage with high per-gene sensitivity—similar to data from single-cell genomics studies—but this has yet to be realized. The lack of such a platform leaves researchers to make difficult decisions about the value of breadth versus sensitivity in their studies, an issue that is further compounded by the lack of historical benchmarks because of the relative novelty of these approaches. Focusing on the 10x Genomics Xenium platform, we attempted to harness the sensitivity of the V1 chemistry (gene panels of up to 480 genes in size) along with the breadth of the Prime chemistry (a predesigned panel of 5,000 genes). Importantly, although the initial off-instrument sample preparation steps of the V1 and Prime chemistries are similar (see Materials and Methods) the on-instrument readout chemistry is substantially different. Thus, we present a method allowing for data generated from both the Prime human 5K panel and a V1 human 480-gene panel on the same slide. The V1 probes are spiked into the 5K probe mixture during sample prep. The probes are then decoded in successive Prime and V1 instrument runs. This results in a combined dataset that captures the breadth of the 5K panel alongside the sensitivity and depth of the V1 custom panel.

## Results

**Figure 1.**
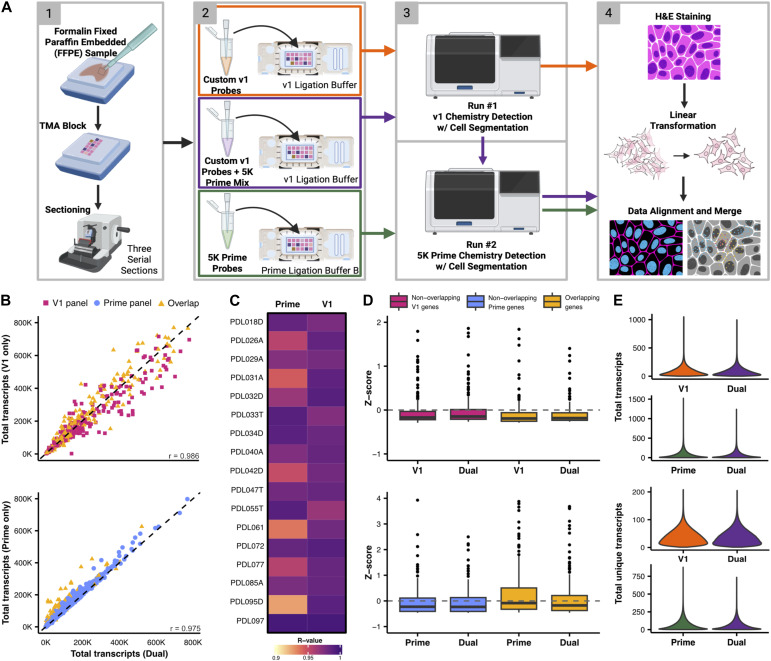
Evaluation of transcript quantification consistency and performance across V1 and Prime panel assays. **(A)** Schematic representation of the study design. **(B)** Scatterplots that show the correlation of raw per-gene transcript counts across all genes detected in the V1 panel (top) and the Prime panel (bottom). The x-axis represents total gene expression from the dual assay (V1 + Prime or Prime + V1), whereas the y-axis represents total gene expression from the solo assay (V1 only or Prime only). Genes are colored by panel specificity: V1-specific genes (pink), Prime-specific genes (blue), and genes overlapping both panels (yellow). **(C)** Heatmap visualizing the R-value derived from correlating the transcript counts from the solo runs with the dual-run transcript counts for 17 infant lung samples. **(D)** Boxplots that display the z-score distribution for three gene sets: the 239 genes overlapping between the V1 and Prime panels, the nonoverlapping V1-specific genes, and a random subset of 239 nonoverlapping Prime-specific genes. Genes are colored by their group: overlapping (yellow), nonoverlapping V1-specific (pink), and nonoverlapping Prime-specific (blue). **(E)** Violin plots of the distribution of total transcripts and total unique transcripts for the three main run types: V1 only, Prime only, and the combined dual run.

Three serial slides of a 17-sample (human lung) tissue microarray (TMA) were prepared using custom V1 probes, human Prime 5K probes, and a combination of the two termed the “dual” slide. The dual slide contained both sets of probes and was loaded on two unique Xenium runs, one V1 and one Prime, to create a dataset of cells with transcriptomics information from both panels (Fig 1A). The V1 solo and Prime solo slides served as technical performance controls for the same 17 samples if only one chemistry was used. For the dual slide, the V1 chemistry was selected to load on the instrument first as it has a shorter runtime. First by comparing the V1 chemistry on the solo versus dual preparation, we found both approaches to segment a similar number of cells (1,111,107 solo versus 1,107,543 dual) and decode a similar number of transcripts (221,439,804 solo versus 218,969,052 dual; Table 1). Importantly, the variance between the solo and dual V1 data was consistent with previously observed levels of slide-to-slide variation between serial sections (Table S1). Turning next to the Prime solo versus the Prime dual preparation (run after the completion of the V1 dual run), we again found a similar number of segmented cells (1,100,358 solo versus 1,115,068 dual) but a drop in the overall number of decoded transcripts (210,218,833 solo versus 187,967,974 dual; Table 1). This suggests running a V1 run before the Prime run modestly decreases the sensitivity of the Prime assay.

**Table 1.** Slide-level transcript detection metrics.

​	Solo V1	Dual V1	Dual 5K	Solo 5K
Number of cells detected	1,111,107	1,107,543	1,115,068	1,100,358
Median transcripts per cell	125	123	100	115
Nuclear transcripts per 100 μm^2^	330.8	322.3	278.8	314.5
Total transcripts	221,439,804	218,969,052	187,967,974	210,218,833

A table containing per slide segmented cell numbers, median transcript count per cell, average transcripts overlapping segmented nuclei per 100 μm^2^ (square micrometer), and the total transcripts detected across the full slide.

Table S1. Slide-level quality control metrics for each run.

**Figure S1.**
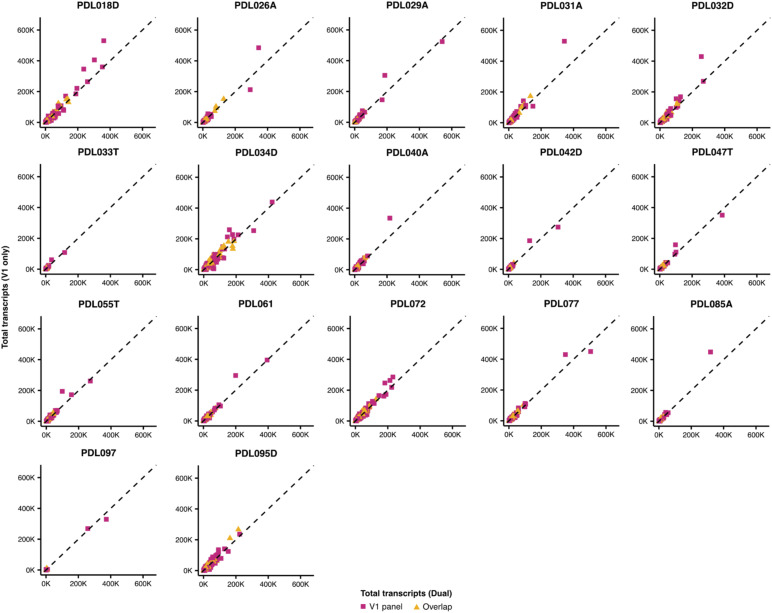
Scatterplots that show the correlation of raw per-gene transcript counts across all genes detected in the V1 panel, split by sample. The x-axis represents the total transcripts for the V1 panel genes, and the y-axis represents the total transcripts for the dual-run genes.

**Figure S2.**
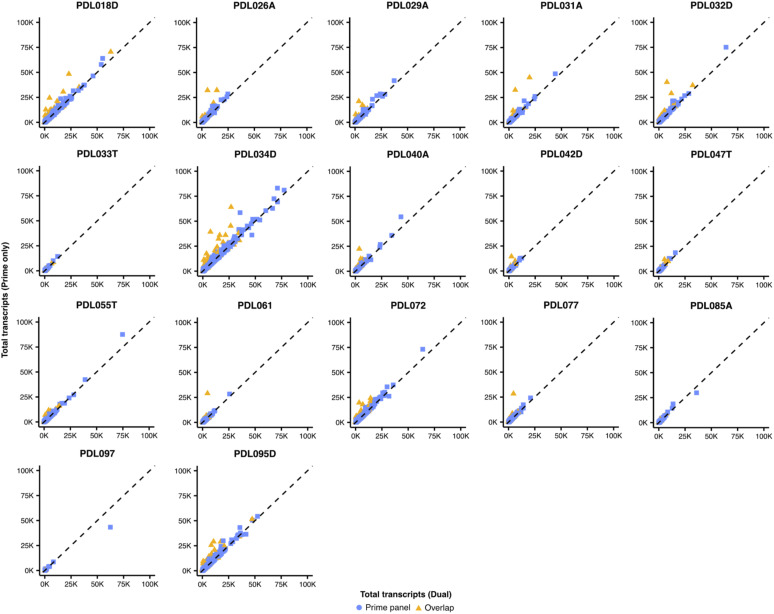
Scatterplots that show the correlation of raw per-gene transcript counts across all genes detected in the Prime panel, split by sample. The x-axis represents the total transcripts for the V1 panel genes, and the y-axis represents the total transcripts for the dual-run genes.

In this study, the V1 panel was designed specifically for use with human lung tissues, whereas the Prime panel was designed to encompass gene expression in a variety of human tissues. The V1 panel detects a total of 480 genes, and the Prime panel detects a total of 5,001 genes. A 239-gene target overlap exists between the two panels allowing us to test for impacts of the same gene being targeted in both panels (Tables S2 and S3). Raw transcript count correlations were examined between the solo and dual slides of each chemistry. The solo V1 and dual V1 runs were highly correlated (r = 0.986; Fig 1B) with no obvious impact on genes overlapping the V1 and Prime chemistries. The Prime solo and dual runs were also highly correlated (r = 0.975; Fig 1B); however, in the genes targeted by both Prime and V1 we observed an exacerbated shift toward lower counts in the dual run compared with the solo run (Fig 1B). Although these correlations were performed at the slide level, each slide contained samples from 17 individuals. At the sample level, high correlation between solo and dual runs is maintained across both chemistries—with none dropping below r = 0.9—although higher sample-to-sample variance is observed with the Prime chemistry (Figs. 1C, S1, S2, and Table S4).

Table S2. Custom v1 panel gene list and design information.

Table S3. Xenium Prime human 5K panel gene list and design information (from 10x Genomics).

Table S4. R-values between each sample across the V1 and Prime gene panels, respectively.

Because these panels were not originally designed to run together, we were concerned that genes targeted with both chemistries may inhibit one another. Sensitivity could be affected by probe-to-probe binding or steric effects of ligation and amplification machinery. To determine whether genes targeted by both V1 and Prime could be driving some differences in performance between the Prime solo and the Prime dual, we quantified sensitivity differences between overlapping and nonoverlapping genes. Briefly, within each run (V1 solo, V1 dual, Prime solo, and Prime dual) we calculated z-scores of expression across the slide. We then examined the z-scores of the 239 overlapping genes compared with a random subset of 239 nonoverlapping genes. If competitive binding drives a difference in sensitivity, we would expect to see a difference in the z-score distribution between the overlapping genes in the solo and dual runs but not the random subset of nonoverlapping genes. Indeed, although we observed similar distributions for V1 solo and dual runs (Fig 1D), we found the z-scores for the Prime solo to be higher than those for the Prime dual—for those genes that overlap between chemistries (Table S5). This is consistent with the shift toward lower counts seen in genes targeted by both chemistries observed in Fig 1B, suggesting that competitive binding between V1 and Prime probes is modestly reducing sensitivity on overlapping genes in the Prime dual run but not the V1 dual run. Along these lines, we also see an increase in the estimated number of false-positive transcripts per cell on the V1 dual (0.3998) when compared to the V1 solo (0.2394), Prime dual (0.1699), and Prime solo (0.1032) (Table S1). This potentially supports the idea that the V1 designed probes are more likely to bind to both their expected and spurious targets.

Table S5. R-values from correlation tests done per sample across the Prime and V1 runs.

**Figure S3.**
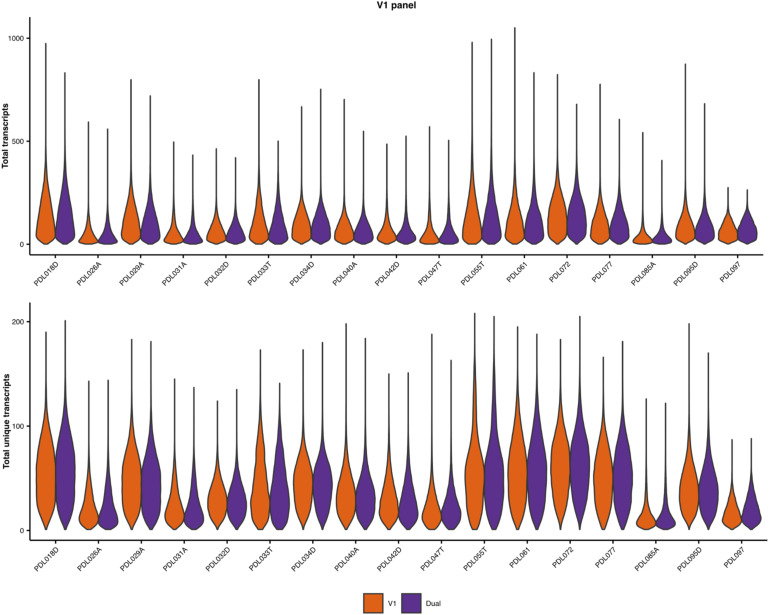
Violin plots that display the distribution of total transcripts and total unique transcripts for V1 and the combined dual run, split by sample.

**Figure S4.**
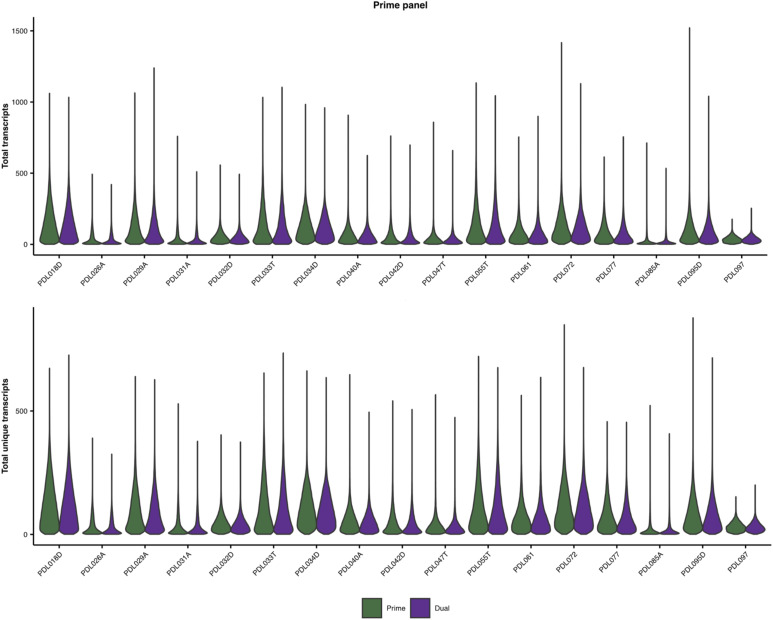
Violin plots that display the distribution of total transcripts and total unique transcripts for Prime and the combined dual run, split by sample.

Another measure of run quality is the total number of transcripts detected on the slide. This metric remained high across all three slides, with detection only dipping under 200,000,000 transcripts on the Prime dual run. When compared within each chemistry, the dual slide shows no major variation in the distribution of total transcripts per cell or total unique transcripts per cell counts (Fig 1E). This remains true on the per-sample level, with multiple samples even outperforming at the cell level on the dual slide when compared to the solo slide in the V1 (Fig S3) and the Prime chemistry (Fig S4).

**Figure 2.**
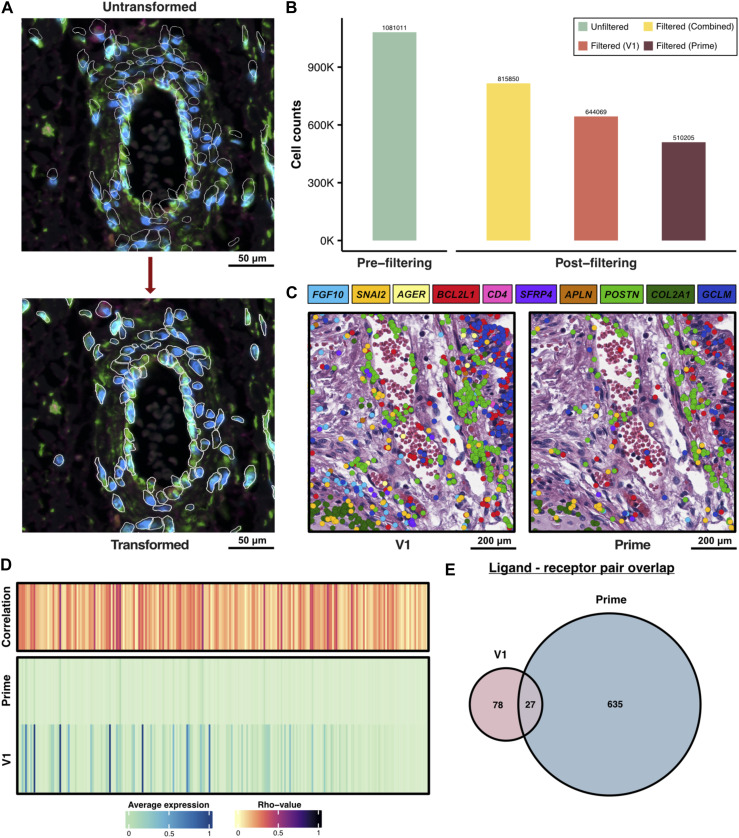
Spatial linear transformation, quality control filtering, and visualization of transcript distribution in dual runs. **(A)** Top panel displays the untransformed dual Prime run image with the original V1 cell segmentation masks (white outlines). The bottom panel displays the result after applying a linear transformation to correct for subtle shifts, successfully aligning the segmentation masks with the underlying cellular morphology. Scale bars are 50 μm. **(B)** Bar chart comparing the initial total cell count (prefiltering) of the dual run with the final cell counts (post-filtering) achieved after applying three layers of filtering to three distinct gene sets: the combined gene panel (yellow), the V1-only gene panel (orange), and the Prime-only gene panel (brown). **(C)** Two adjacent panels show the same histological field of view, with underlying morphology by hematoxylin and eosin staining. The left panel overlays the spatial distribution of transcripts detected by the V1 panel, and the right panel overlays the transcripts detected by the Prime panel. Both images display the expression of 10 selected genes, with their respective transcripts colored according to the legend above. Scale bars are 200 μm. **(D)** Heatmap quantifying the relationship between the V1 and Prime panels by showing the average expression of 239 overlapping genes and their expression correlation (Rho-value). **(E)** Proportional Venn diagram quantifying the overlap of LR pairs detected between the V1 and Prime panels in epithelial–mesenchymal proximal cell pairs on the dual slide. Circle areas are proportional to the number of LR pairs detected in each panel. Prime detected substantially more LR pairs than V1, with the majority unique to Prime, a smaller subset unique to V1, and 27 pairs shared between both panels, highlighting the limited but existing overlap in LR pair detection across the two panel chemistries.

**Figure S5.**
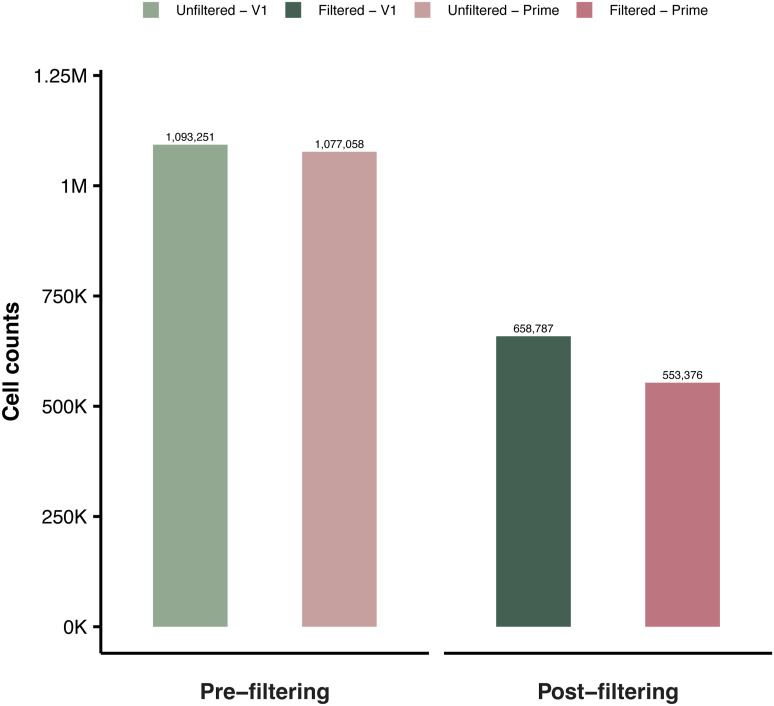
Bar chart comparing the initial total cell count (prefiltering) of each solo slide (V1—green; Prime—pink) with the final cell counts (post-filtering) achieved after applying the same filtering thresholds in accordance with those used for the dual slide: number of transcripts per cell >= 50, number of unique transcripts per cell >= 5, overall cell area >= 5 & <= 140, nucleus area >= 3.

The key benefit of running both Xenium chemistries on the same slide is for co-detection of the higher sensitivity V1 and the broader reaching Prime panel in the exact same cells. Although an adjacent section provides a decent proxy, it is still a 5-μm offset that then relies on the alignment of nonmatched nuclei and is further complicated by the fact that cells exist in a 3D space with cytoplasmic overlap at that 5- μm scale. In order to combine data from the two runs of the dual slide, the transcripts first need to be put in a common cell coordinate system. In order to do this, a single segmentation mask must be applied to both runs. In this case, we chose the mask from the V1 run. However, a linear transformation had to be applied to the original V1 run segmentation masks to align them with the slightly shifted cell placement from the Prime run (Fig 2A). The shift occurred because of a small technical variation that can occur when exchanging the dual slide from a V1 Xenium cassette and into a Prime Xenium cassette, as well as any slight changes to the exact placement of the cassette on the instrument stage during the second run leading to very small offsets. We expect this value to be variable and will thus require some amount of slide-specific correction on any future slides run using this method. All of the alignment adjustments were made within the validated 10x Genomics’ software ecosystem, meaning that the results are expected to be reproducible on additional slides, sample types, and panels. After the linear transformation, the detected transcripts falling within those bounds could be united under the same cell id for both the V1 and Prime runs, creating a combined gene set for each segmented cell on the dual slide. Cells were then filtered using the following parameters to eliminate cells that would not meet normal thresholds for downstream analysis: n_counts >= 50, n_genes >= 5, cell_area >= 5 & <= 140, nucleus_area >= 3. These cutoffs were used to filter the 1,081,011 cells segmented on the dual slide using transcripts only from the V1 panel, transcripts only from the Prime panel, and transcripts from the combined dataset. Using the combined transcripts left a greater number of retained cells, post-filtering, compared with the V1 or Prime panel transcripts alone (Fig 2B). 75.5% of cells met the thresholds in the combined dataset, 265,161 cells lost and 815,850 retained. 59.6% of cells met the thresholds using V1 transcripts only, 436,942 cells lost and 644,069 retained. Less than half of the cells at 47.2% met the thresholds using Prime transcripts only, 570,806 cells lost and 510,205 retained. We applied the same downstream analysis parameters to the V1 solo and Prime solo slides and observed a consistent pattern of cell retention: 60.3% retained in V1, and 51.4% retained in Prime (Fig S5), thus supporting the idea that data from the combined panels allowed for a higher number of cells to meet quality thresholds and be included in downstream analysis. This trend of the dual dataset retaining the most cells and the Prime panel alone retaining the least was consistent across all samples on the dual slide, with one exception in PDL034D where the Prime panel alone retained 149 more cells than the V1 panel alone with both still falling short of the combined panel (Table S6).

Table S6. Cell counts per sample pre/post-filtering on the dual slide.

**Figure S6.**
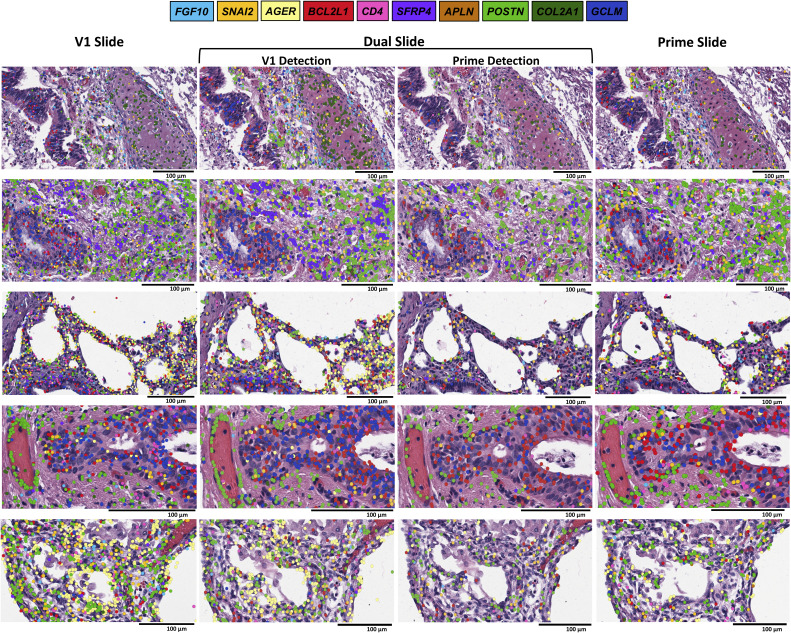
Additional matched histological fields of view showcasing the spatial distribution of V1 gene detection on the V1-only slide (leftmost column), V1 gene detection on the dual slide (middle-left column), Prime gene detection on the dual slide (middle-right column), and Prime gene detection on the Prime-only slide (rightmost column), with underlying hematoxylin and eosin morphology. All images display the expression of 10 selected genes that span cell-type lineage, with their respective transcripts colored according to the legend above. Scale bars are 100 μm.

**Figure S7.**
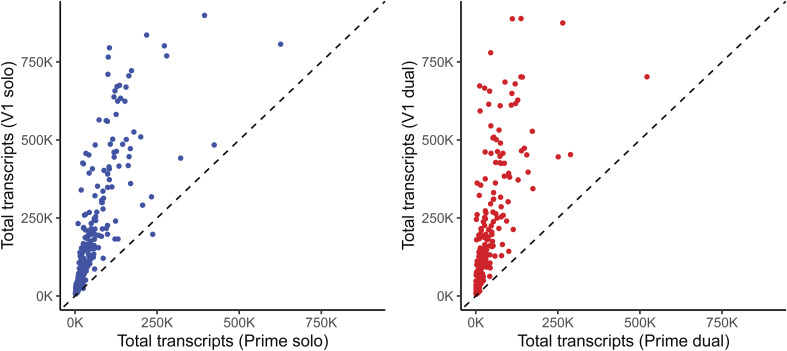
Scatterplots that show the correlation between total detected transcript counts between V1 and Prime chemistries run alone and then both chemistries on the dual slide. Each point represents the total detected transcript count for one of the 239 overlapping genes.

Given the shared 239 genes in the V1 and Prime panels, we were able to perform cross-chemistry comparisons. First by performing a qualitative assessment of 10 representative overlapping genes, we observed similar expression profiles for the V1 and Prime chemistries across differing architectures—albeit with lower transcript counts in the Prime chemistry (Figs 2C and S6). Nevertheless, we observe only modest correlations between the V1 and Prime expression levels on the dual (see Materials and Methods; Fig 2D and Table S7). Indeed, the low correlation is likely driven by the differences in sensitivity between the two chemistries (Fig 2D). A large portion of cells in the dual-run dataset have a low dynamic detection range in Prime, with many observed zeros. Furthermore, when we compared the overall slide-level correlation of transcript counts for the 239 shared genes, we see very similar trends between the V1 and Prime chemistries in both the solo and dual slides (Fig S7). Together, this suggests the modest correlations between the dual V1 and Prime data are driven by technical differences inherent to the chemistry—rather than the dual-run approach. However, the true value of this integrated approach is the ability to couple a targeted gene panel—focused on cell-type annotation and known biological markers—with a more discovery-oriented approach. Although this study was not designed to showcase the breadth of these possibilities, an example of such is illustrated by the ligand–receptor (LR) analysis. With the additional genes available on the dual run, we investigated what cell–cell interactions could be detected within each chemistry. Using lineage-level cell-type annotations, we focused on epithelial cell ligands in neighboring proximity (≤10 μm) to a mesenchymal cell harboring a compatible receptor, using a curated list of known LR pairs. A fivefold increase in unique LR pairs was detected in the Prime panel compared with the V1 panel alone (Fig 2E and Table S8). We detected 78 unique LR pairs in the V1 panel, 635 unique pairs in the Prime panel, and 27 shared pairs in both panels. In order to profile the same diversity of targets needed to capture this number of unique LR pairs, our entire 480-gene V1 panel would need to be dedicated solely to LR analysis, thus highlighting the potential for the discovery of novel interactions between cells when combining the Prime panel with the cell-typing capabilities of a custom V1 panel. The breadth of possible analyses will continue to grow as more granular cell types are annotated, enabling a deeper biological understanding of the tissue profiled under the dual chemistry.

Table S7. Full-slide transcript counts, average expression, and correlation values across chemistries for all overlapping genes.

Table S8. Table of known LR pairs queried in our analysis and if they were detected in either chemistry on the dual slide.

## Discussion

There are several limitations to the work described in this study. First, the sample set contained in this article is limited to a single group of 17 unique pulmonary samples. We are not able to draw any conclusions about the replicability of the results observed across other tissue types. We were able to use the two adjacent sections prepared with the V1 and Prime solo chemistries to judge performance, but even those are imperfect proxies. Secondly, the fact that V1 regularly outperforms Prime for sensitivity on our lung samples can make the comparison a bit difficult to judge performance between the solo chemistries and the dual. According to the annotations given by 10x Genomics, 392 of the genes found on the 5K panel are explicitly labeled to be found in “lung” cell types (Table S3). There are likely many other genes that are relevant to the lung tissue in general. However, many of the higher expressing genes that are critical for cell typing (e.g., *SFTPC, SCGB3A2, COL1A1*) are noticeably absent to preserve the optical detection space in order to accommodate the higher number of targets overall in Prime. We attempted to mitigate this bias by looking largely at the dual slide as a single dataset rather than assessing each chemistry separately. This is also largely the reason that the V1 chemistry results in better filtering, so the relative comparisons with the dual are not as easily discerned.

Although we only provide one example of the opportunities provided by having a combined dataset, we believe there are significant opportunities for discovery. These range from obvious additional analyses, such as extended LR analysis carried out on the background of high-fidelity cell typing, to more nuanced approaches, such as deriving project-specific imputation approaches—using the V1 data to impute the missing Prime or vice versa. A current barrier for widespread implementation of this method is the increased cost per slide. To prepare the data in this article, the dual prep required the purchase of both a V1 Xenium Sample Prep Reagents kit (1000460; 10x Genomics) and a Xenium Prime 5K Human Sample Prep Reagents kit (1000671; 10x Genomics), doubling or tripling the cost depending on the V1 panel chosen. Indeed, an imputation approach would enable researchers to only perform the dual profiling on a single slide containing a representative subset of samples from a larger cohort. This route could alleviate the potentially cost-prohibitive nature of running the dual assay for every slide and should be investigated further. For precious or archived samples where a maximum amount of data is desired without access to an additional slide, the extra cost may be warranted to enable deeper analyses. This study serves as a proof of concept that such techniques are possible, but the final cost/benefit analysis of the dual-chemistry approach will be highly project-dependent.

Finally, although the sensitivity benefits of small custom probe-based studies have been appealing, they suffer from difficulties with cross-study integration. Since the advent of genomics, the field has benefited from mappings to common references that allowed large post hoc meta-analyses. However, with small curated panels of genes, even studies within the same organ system may have limited overlap—depending on the motivation of the research. This severely hinders the ability to integrate disparate datasets leading to a diminished future utility. In contrast, this approach provides a stable common reference (the Prime chemistry) without sacrificing the sensitivity of key genes. This shared target space will allow cross-study integration providing future value for the scientific community. Although not explicitly tested in this study, we strongly believe that the dual-assay approach will allow co-detection of on-instrument protein panels. This is based on the proven compatibility of the cell morphology stains used for cell segmentation, which are detected in the same style of imaging cycle, thus providing an additional layer of information all contained in the same sample section.

The ability to co-detect tissue- and sample-specific V1 panels alongside Prime 5K panels in the same cells without signal dropout opens the door to greater biological understanding. It provides detection in a large shared 5,001-gene base on which large datasets across institutions can be aligned, while also allowing for individualized V1 panels to target specific questions or discoveries. Having greater combined histological and expression context is the central goal of Xenium spatial transcriptomics technology, meaning that having the breadth of the Prime 5K panel matched with the depth and sensitivity of a V1 panel maximizes that potential as far as the current technology allows.

## Materials and Methods

### Sample selection

Samples included in this study were archived formalin-fixed paraffin-embedded (FFPE) tissues from human lungs. Samples were collected under STUDY00003664 at Seattle Children’s Hospital and from the Human Infant Lung Repository, a resource supported by the Biodevelopmental Origins of Lung Disease Center at Vanderbilt University School of Medicine. The collection and use of these tissues were reviewed and approved by the Institutional Review Board for Human Subject Research at Seattle Children’s Hospital and the Vanderbilt University Institutional Review Board, respectively.

A section was taken from the face of each sample block for standard hematoxylin and eosin staining. A team with expertise in pediatric lung pathology selected a 3 mm ✕ 3 mm target location for the region of interest. Archived samples contained within this study were collected, processed, and embedded within the last 5 years (n = 10), 10 years (n = 6), or 20 years (n = 1, PDL072). Because of the precious nature of these samples and the robust performance of Xenium with archived samples, RNA quality (DV200) scoring was not performed.

### TMA construction

The Xenium capture area is 10.45 mm ✕ 22.45 mm. The use of a TMA allows for the more precise placement of multiple tissue samples within that area. The FFPE TMA block was designed in a 3 ✕ 6 pattern of 17 3-mm cores, with the top left core space remaining empty as an orientation marker.

A custom 3-mm square tip was created by the Arizona State University Instrument Design and Fabrication Core to fit the commercially available Quick-Ray Manual Tissue Microarrayer and allow us to produce square cores. The TMA was constructed by placing each core individually, tissue face down, on a double-sided adhesive tape in a paraffin block mold (7, 8). The entire mold is warmed to 37 °C, and a P1000 is used to dispense melted paraffin wax in the junctions between cores. More wax is poured over the back of the block to fill in the remaining space and adhered in a plastic cassette. Once solidified, the block was extracted from the mold, the tape was removed, and the block was stored at 4°C until sectioning.

### Sectioning and sample placement

The Leica RM2135 microtome and surrounding area were cleaned with RNase AWAY and 70% isopropanol before sectioning. The TMA block was rehydrated using nuclease-free water and then cut with a DURAEDGE Low-Profile PTFE blade at 5 μm. Sections were floated on a 40°C heated water bath filled with nuclease-free water, to help remove wrinkles. Three serial sections were taken, and each was placed on an individual Xenium slide. Slides were dried overnight at room temperature, then baked the next day for 3 h at 37°C. After this incubation, slides were stored in a sealed desiccator at room temperature for 14 d before Xenium sample preparation.

### Gene panel design

All Xenium runs, V1 or Prime 5K, require the use of a gene panel. A total of 480 genes were included in our V1 custom panel. These genes were chosen to cover expression in the human lung and enable cell-type identification based on single-cell analysis data (9) and Xenium analysis data (10). The Xenium Prime 5K Human Pan Tissue and Pathways panel contains 5,001 genes and was designed by 10x Genomics to cover expression in a variety of human tissues. 239 genes are present on both our custom V1 panel and the predesigned Prime panel (Tables S2 and S3). Probe sequence files are available for download on GEO under accession GSE315411.

### Custom probe resuspension

New tubes of our custom V1 panels were used for this experiment. This facilitated the resuspension of the lyophilized probes in a lower volume than normal, which is required to properly spike into the Prime panel and maintain relative concentration. This also allowed both the V1-only slide and the dual slide to be prepped with the same lot of probes to maintain consistency.

6ZAAWG, our custom base panel, was ordered in a 16-reaction quantity and resuspended in ⅓ of the normal volume TE buffer (233.33 μl versus 700 μl, CG000749; 10x Genomics). 6ZX7JP and 43R3RN, our custom add-on panels, were ordered in 16-reaction quantities, and each was resuspended in ⅙ of the normal volume TE buffer (116.67 μl versus 700 μl, CG000749; 10x Genomics). In a normal V1 run, these 50-gene panels are still resuspended at double the concentration of a single add-on in order to add both to 6ZAAWG while maintaining the 33 μl add-on volume (16.5 μl each).

### Xenium sample preparation

Xenium relies on intact in situ RNA for binding, so all workstations and equipment are cleaned using RNase AWAY (53225-514; VWR) followed by 70% isopropanol. All reagents, including water, were molecular-grade nuclease-free to further preserve RNA quality.

The V1-only slide underwent deparaffinization and decrosslinking according to the 10x Genomics Demonstrated Protocol CG000578 without alteration. This slide underwent probe hybridization using the following probe mix recipe: 315 μl Xenium Probe Hybridization Buffer, 144 μl TE buffer, 33 μl 6ZAAWG, 16.5 μl 2X 6ZX7JP, 16.5 μl 2X 43R3RN. Probe hybridization occurred at 50°C for 19 h. Ligation, amplification, cell segmentation, autofluorescence quenching, and nuclei staining were performed according to the 10x Genomics User Guide CG000749 without alteration. The slide was stored at -20°C in 30% glycerol solution in PBS for 18 d following sample prep completion before loading onto the Xenium Analyzer.

The Prime-only slide underwent deparaffinization and decrosslinking according to the 10x Genomics Demonstrated Protocol CG000578 without alteration. This slide underwent probe hybridization using the following probe mix recipe: 94.5 μl Probe Hyb Buffer B, 10.5 μl TE buffer, 52.5 μl Xenium 5K Hu PTP Panel Probes. Probe hybridization occurred at 50°C for 16 h. Priming, RNase treatment, polishing, ligation, amplification, cell segmentation, autofluorescence quenching, and nuclei staining were performed according to the 10x Genomics User Guide CG000760 without alteration. The slide was stored at −20°C in 30% glycerol solution in PBS for 22 d following sample prep completion before loading onto the Xenium Analyzer.

The dual-chemistry slide underwent deparaffinization and decrosslinking according to the 10x Genomics Demonstrated Protocol CG000578 according to the Prime chemistry directions. This slide underwent sample preparation according to the 10x Genomics User Guide CG000760 Prime chemistry with the following alterations. The probe hybridization mix recipe was 94.5 μl Probe Hyb Buffer B, 52.5 μl Xenium 5K Hu PTP Panel Probes, 5.25 μl 3X 6ZAAWG, 2.63 μl 6X 6ZX7JP, 2.63 μl 6X 43R3RN. Probe hybridization occurred at 50°C for 16 h. For the ligation steps, Xenium Ligation Buffer (2000391; 10x Genomics) from the V1 chemistry kit was used instead of Ligation Buffer B (2001233; 10x Genomics) from the Prime chemistry kit. At the end of the prep, the slide was stored at −20°C in 30% glycerol solution in PBS for 18 d before loading onto the Xenium Analyzer for the first run.

### Xenium Analyzer imaging

Detailed instructions for instrument configuration, consumables, and buffer preparation can be found in the 10x Genomics User Guide CG000584. After loading and manual area selection on a low-resolution full-slide image, the Xenium Analyzer fully automates the collection of fluorescent punctum data through rounds of fluorescent probe binding, imaging, and stripping. Each potential RNA punctum location is then decoded and labeled by gene ID, according to the fluorescence pattern across imaging channels and cycles. All image fields of view (4,240 ✕ 2,960 pixels) are then stitched together using the nuclei-stained DAPI image with RNA transcript locations overlaid in the x-y-z coordinate system of the images. Onboard instrument analysis provides quality metrics for each detected transcript based on decoding confidence from the image cycle signals. Additional fluorescent images are taken at the end of the run to enable cell segmentation based on cell nuclei, cell boundary, RNA, and interior protein staining. All data in this article were collected on instrument software version 3.4.1.0 and onboard analysis version xenium-3.3.0.1.

The Xenium Analyzer instrument performs data acquisition for a maximum of two slides per run. First loaded were the V1-only and the dual slide for a V1 decoding run. Cell segmentation was performed on this run. After run completion, slides were removed from the instrument. The post-run buffer in the slide cassette wells was removed and replaced with fresh 1,000 μl PBS-T. The V1 slide was stored at 4°C until post-run histology staining was performed. The dual slide was stored at 4°C for ∼12–16 h before reloading onto the instrument for a second run.

The second batch of slides loaded was the Prime-only and the dual slide for a Prime 5K decoding run. Cell segmentation was performed on this run. After run completion, slides were removed from the instrument. The post-run buffer in the slide cassette wells was removed and replaced with fresh 1,000 μl PBS-T. Both slides were stored at 4°C until post-run histology staining was performed.

### Post-Xenium histology

All three slides underwent quencher removal in sodium hydrosulfite according to the 10x Genomics Demonstrated Protocol CG000613. Immediately after, the slides were hematoxylin- and eosin-stained according to the following protocol: DI water (2 min), Mayer’s hematoxylin (2 min; MHS16-500ML; MilliporeSigma), DI water (x3, 1 min each), bluing solution (1 min; CS70230-2; Agilent), DI water (1 min), 95% ethanol (3 min), eosin Y (45 s; NC2101164; Thermo Fisher Scientific), 95% ethanol (1 min), 100% ethanol (x2, 30 s each), xylene (x2, 3 min each). Coverslipping was performed using ∼150 μl Leica Mounting Media (NC1109222; Thermo Fisher Scientific) and #1.5 50 × 24 mm cover glass (16002-264; VWR). Mounting media were cured for at least 30 min at room temperature. Histology images were taken on a 40× (20× objective with doubler inserted) Leica Biosystems Aperio CS2.

### Cell segmentation

Cell segmentation was performed on the Xenium Analyzer instrument using the onboard multimodal cell segmentation algorithm with default parameters (onboard analysis version xenium-3.3.0.1).

### Image registration

Image registration between the DAPI images of the V1 and Prime dual runs was performed in Xenium Explorer software (version 4.1.0). Briefly, the DAPI image from the V1 run was aligned to that of the Prime run through a linear transformation based on the placement of anchors on corresponding landmarks in both images.

### Cell mask transformation

Cell masks from the V1 run were transformed into the coordinate system of the Prime run using the linear transform produced by image registration and the import-segmentation command within Xenium Ranger (version 4.0) (11). Final fine-scale adjustments to the alignment were made using a custom Python script. After these adjustments were made, import-segmentation was run again to update the transcript assignments with this final alignment. All software necessary to perform the alignment is published and validated by 10x Genomics. Alignment can be performed on a workstation meeting minimum requirements for Xenium Explorer as specified by 10x Genomics (12).

### Quality control—upstream filtering

Raw Xenium output resulted in transcript and cell metadata information. Each transcript file (per slide) contained metrics for each transcript, including spatial coordinates, cell-level information (cell_id), quality value (QV), and nuclear overlap (overlaps_nucleus). We used Seurat (13) to create objects with this information and filtered transcripts to remove any that did not meet quality parameters: QV < 20, overlaps_nucleus = 0, and cell_id = UNASSIGNED. We created gene matrices based on the remaining transcripts that were partitioned into a segmented nucleus, and with those matrices and metadata information, we generated Seurat objects per each slide that were merged into a singular object.

### Panel-specific object creation

One Seurat object was made with the output information from the V1 solo run and the dual run with V1 segmentation, and one with the Prime solo run and the dual run with Prime segmentation. The dual V1/Prime run contains the same cells across both iterations. However, Xenium uses randomly generated strings for cell labels, and to ensure that the cells are correctly matched, the unique string was generated per row of metadata across the run with the V1 segmentation and the Prime segmentation to match cells accordingly post-linear transformation. In order to successfully combine the gene matrices across the V1 and Prime panels, unique identifiers were appended to each feature name in the transcript files, which would allow the matrices to be combined into one.

We then used Scanpy (14, 15) to create an AnnData object with only the dual run, but both segmentation types, from these adjusted transcript data. We calculated various metrics (number of counts/genes per cell) with respect to the V1, Prime, and the combined panel genes. We then created two following AnnData objects—one with the V1 and Prime genes, respectively. Cells from each object were retained according to the following parameters: n_counts >= 50, n_genes >= 5, cell_area >= 5 & <= 140, nucleus_area >= 3.

### Clustering and lineage annotation

To do any clustering, we required the use of a graphical processing unit, given the magnitude of data. We used a container with rapids_singlcell (16)—single-cell Python library (Scanpy v1.8.1) to perform clustering and dimensionality reduction across all three panel-specific objects. Each object was clustered with 20 PCs and 15 nearest neighbors.

We conducted lineage-level cell annotation that was anchored on the cluster labels from the dual run from above, and we used a combination of canonical genes (9, 17, 18) per general lineage to stratify each cluster into four sublineages: epithelial (*EPCAM, SCGB3A2, KRT5, MUC5B, AGR3, SFTPC, AGER, KRT8*), endothelial (*PECAM1, EPAS1, VWF*), mesenchymal (*COL1A2, DCN, LUM, ACTA2*), and immune (*PTPRC, BANK1, CD1C, TRAC, IL-7R, FCER1G, JCHAIN, S100A8, MARCO*).

### Cellular proximity

We extracted epithelial and mesenchymal cell centroids across all samples in the dual run. For every unique epithelial cell, we identified its nearest mesenchymal cell neighbor using k-nearest neighbor search (k = 1) implemented in RANN::nn2 (19) within a Euclidean distance of ≤ 10 μm (10). We parallelized this workflow across 24 cores using doParallel (20).

### LR analysis

To determine which LR pairs from the database were detectable within each Xenium panel, we assessed gene coverage before expression analysis. We loaded the human LR pair database from CellTalkDB (21) and extracted all unique ligand and receptor gene symbols. We identified direct matches between LR gene symbols and panel gene lists for the V1, Prime, and dual panels. To account for genes listed under alternate or legacy HGNC symbols, we performed a two-pass BioMart (22) query against the Ensembl (23) database. In the first pass, we retrieved all known synonyms for each LR gene. In the second pass, we queried panel genes without a direct match as potential synonyms for LR genes. We considered a LR pair complete if both the ligand and receptor were represented in the panel, either by direct symbol match or by alias resolution.

For each proximal epithelial–mesenchymal cell pair, we extracted raw transcript counts from the Seurat count matrix. We stripped panel-specific unique gene suffixes (“-v1-prime” for the 480-gene panel; “-prime-with-v1-seg” for the 5,000-gene panel) to match gene symbols to the LR database. We recorded a LR interaction if the ligand had raw counts >0 in the epithelial cell and the cognate receptor had raw counts >0 in the paired mesenchymal cell, treating epithelial cells as ligand-expressing sender cells and mesenchymal cells as receptor-expressing receiver cells. We performed the analysis independently for each panel and identified interactions detected in both panels as overlapping hits.

### Correlations

In this study, we compared the gene expression of 239 overlapping genes across the V1 and Prime panels using Spearman’s correlation test. To do this, we aggregated the expression across all cells per gene into one value. From there, the correlation was done between each shared gene across both panels.

## Protocol Availability

Step-wise methods can be found on protocols.io at DOI: dx.doi.org/10.17504/protocols.io.5qpvodwm7g4o/v1.

## Code Availability

Custom R and Python scripts for this project are available on GitHub at https://github.com/Banovich-Lab/xenium_v1_prime_comparison.

## Supplementary Material

Reviewer comments

## Data Availability

Raw and processed data are deposited at GEO under accession GSE315411.
