# Supplementary material for: Fishing with two lines: a hybrid approach to spatial transcriptomics discovery
Source: Life Sci Alliance. 2026 Jul 27;9(10):e202603690. doi: 10.26508/lsa.202603690 (PMC13404378; doi:10.26508/lsa.202603690)
Supplement: Reviewer comments [file LSA-2026-03690_review_history.pdf]

Fishing with Two Lines: A Hybrid Approach to Spatial Transcriptomic Discovery

Arianna Williams-Katek, Saahithi Mallapragada, Evan Mee, Brandon Fischer, Annika Vannan, Laurie Eldredge, Gail Deutsch, Jonathan Kropski, Jennifer Sucre, and Nicholas Banovich
DOI: https://doi.org/10.26508/lsa.202603690

Corresponding author(s): Arianna Williams-Katek, Translational Genomics Research Institute and Nicholas Banovich, Translational Genomics Research Institute

Review Timeline:

Submission Date:	2026-03-10
Editorial Decision:	2026-04-06
Revision Received:	2026-06-01
Editorial Decision:	2026-06-30
Revision Received:	2026-07-13
Accepted:	2026-07-15

Scientific Editor: Tim Fessenden

Transaction Report:

(Note: With the exception of the correction of typographical or spelling errors that could be a source of ambiguity, letters and reports are not edited. The original formatting of letters and referee reports may not be reflected in this compilation.)

April 6, 2026

Re: Life Science Alliance manuscript #LSA-2026-03690

Ms. Arianna L. Williams-Katek
Translational Genomics Research Institute
445 N 5th St
Phoenix, AZ 85004

Dear Dr. Williams-Katek,

Thank you for submitting your manuscript entitled "Fishing with Two Lines: A Hybrid Approach to Spatial Transcriptomic Discovery" to Life Science Alliance. The manuscript was assessed by expert reviewers, whose comments are appended to this letter. We invite you to submit a revised manuscript that addresses the reviewers' main concerns.

As you will see, reviewers uniformly appreciated this proof-of-concept demonstrating successful application of Visium Prime5K and V1 probes to the same sample. Reviewers 1 and 3 felt the manuscript would benefit by stronger evidence for the biological insight made possible by this approach, especially in view of its cost and potential complexity. We agree that the suggestion by Reviewer 3 should be implemented towards this goal. Reviewer 2 made two important technical points that should be addressed (points 1 and 2). Reviewer 3 also made several suggestions to improve clarity of the figures (Figures 2 and 3) which we invite you to consider. Finally we suggest you consider providing the target sequences and their overlap for Prime5K and V1 probes (as supplemental data) for greater transparency and confirmation that the high resolution from Prime5K is not compromised by probe overlap. However, including that dataset is left to your discretion.

I would be happy to discuss the revision in more detail via email or phone/videoconferencing. Please let me know which option you prefer, if any.

To upload the revised version of your manuscript, please log in to your account: https://lsa.msubmit.net/cgi-bin/main.plex

You will be guided to complete the submission of your revised manuscript and to fill in all necessary information. Please get in touch in case you do not know or remember your login name.

The typical timeframe for revisions is three months. Please note that papers are generally considered through only one revision cycle, so strong support from the referees on the revised version is needed for acceptance.

While you are revising your manuscript, please also attend to the below editorial points to help expedite the publication of your manuscript. Please direct any editorial questions to the journal office. When submitting the revision, please include a letter addressing the reviewers' comments point by point.

Thank you for this interesting contribution to Life Science Alliance. We hope that the comments below will prove constructive as your work progresses, and we are looking forward to receiving your revised manuscript.

Sincerely,

Tim Fessenden
Scientific Editor
Life Science Alliance

A. THESE ITEMS ARE REQUIRED FOR REVISIONS

-- A letter addressing the reviewers' comments point by point.

-- An editable version of the final text (.DOC or .DOCX) is needed for copyediting (no PDFs).

-- High-resolution figure, supplementary figure and video files uploaded as individual files: See our detailed guidelines for preparing your production-ready images, https://www.life-science-alliance.org/authors

-- Summary blurb (enter in submission system): A short text summarizing in a single sentence the study (max. 200 characters

including spaces). This text is used in conjunction with the titles of papers, hence should be informative and complementary to the title and running title. It should describe the context and significance of the findings for a general readership; it should be written in the present tense and refer to the work in the third person. Author names should not be mentioned.

-- By submitting a revision, you attest that you are aware of our payment policies found here: https://www.life-science-alliance.org/copyright-license-fee

B. MANUSCRIPT ORGANIZATION AND FORMATTING:

Full guidelines are available on our Instructions for Authors page, https://www.life-science-alliance.org/authors

We encourage our authors to provide original source data, particularly uncropped/-processed electrophoretic blots and spreadsheets for the main figures of the manuscript. If you would like to add source data, we would welcome one PDF/Excel-file per figure for this information. These files will be linked as supplementary "Source Data" files.

IMPORTANT: It is Life Science Alliance policy that if requested, original data images must be made available. Failure to provide original images upon request will result in unavoidable delays in publication. Please ensure that you have access to all original microscopy and blot data images before submitting your revision.

Reviewer #1 (Comments to the Authors (Required)):

This manuscript presents a technically well-executed dual-chemistry spatial transcriptomics approach that combines Xenium V1 and Prime panels on the same tissue section. The benchmarking is thorough and the effort to bridge sensitivity and plex is appreciated. However, I have several concerns regarding the practical utility, scalability, and biological added value of this approach, especially in a field that is rapidly evolving toward higher plex and improved sensitivity within single assays.
My concerns are:
Scalability: The current workflow requires sequential runs and dual chemistry preparation on the same slide. It is unclear how this approach could be scaled to larger studies or cohorts, particularly in high-throughput settings.
Cost: The method requires both V1 and Prime reagent kits and multiple instrument runs. This represents a substantial increase in cost per sample, which may limit adoption. A more quantitative discussion of cost vs. benefit would be valuable.
Compatibility with protein detection It would be important to clarify whether this dual workflow is compatible with protein co-detection, which is increasingly a key feature of spatial platforms.
Limited biological validation: The method is only demonstrated in one tissue type (human lung). It is unclear whether the same performance and trade-offs would hold across other tissues or disease contexts.
Computational and technical overhead: The approach introduces additional steps (image registration, segmentation alignment, dataset integration), which increase computational burden and potential sources of variability. A discussion of robustness and reproducibility of these steps would strengthen the manuscript.
Unclear biological added value: While the technical integration is convincing, the biological advantage remains somewhat underdeveloped. The manuscript would benefit from a clearer demonstration of analyses or insights that are uniquely enabled by the dual approach and could not be achieved with existing high-plex or high-sensitivity methods alone.
In summary, this is a careful and well-benchmarked technical study. However, given the increased cost, complexity, and unclear scalability, the practical impact of this approach remains uncertain in the context of rapidly advancing spatial transcriptomics technologies. While the technical integration is convincing, the biological advantage is insufficiently demonstrated. It is not entirely clear what new biological insights or discoveries are uniquely enabled by the dual approach, beyond what could be achieved with a well-designed targeted panel or a high-plex panel alone. The examples provided are illustrative but do not establish a compelling use case where dual chemistry is necessary rather than incremental. The authors should more clearly articulate and substantiate the unique biological value of this approach, and make a stronger case for why the added complexity is justified over existing alternatives.

Reviewer #2 (Comments to the Authors (Required)):

Summary:
The authors present a clever method to combine the high-sensitivity Xenium assay with high-breadth the Xenium assay, overcoming some of the trade-offs between targeted and discovery-based assays. This paper is very thorough and overall convinces me that the drawbacks in sensitivity associated with combining assays are relatively minimal, so users who cannot afford to trade off these elements should consider the approach. However, there are a few missing pieces to this manuscript that need to be addressed before recommending for publication.

1. Aside from concordance between the solo vs. dual approach in transcript detection sensitivity (e.g. counts), it is important to

consider whether localization of gene expression is broadly in agreement between the solo vs. dual assays. The authors do not show any images that represent transcript localization between the solo vs. dual approach. The authors visually compare the Prime vs. V1 results (dual vs. dual) across a few relatively small fields of view across 10 genes, which suggests a drastic drop in sensitivity in the Prime assay. The reviewer suggests that the authors show solo vs. dual, and tissue- or slide-level figures across more than just 10 genes to represent the level of agreement in transcript localization. This would be more interpretable than the clustering UMAP results that are not spatially localized as shown in Figure 3. An additional/alternative showcase could be to display the spatial representation of UMAP clusters on the dual vs. solo slides.
2. While the Prime assay has 40-60% reduced sensitivity on a per-gene basis in comparison to the low plex assay, it is very surprising that the authors are seeing a drop in total detected transcripts in the Prime assay (across 5,000 genes) compared to the V1 assay. This brings into question either the Prime assay execution, tissue quality, or relevance of the 5K panel to lung tissue. Can the authors comment on this? Things to consider are how old the archived FFPE block is (this is not stated), what its DV200 score is, and how many genes from the 5K panel are expected to be relevant to the tissue type.
3. Can the authors comment on compatibility of the 5K and low plex probes themselves? For example, is there cross-talk or complementary sequences in the barcode regions of the probes that could cause the Prime and low plex probes to spuriously bind to each other and amplify?
4. Can the authors comment on how false positive rates are affected by the dual run?
5. Is the information from 10X for combining 5K + low plex assays (particularly the oligo sequence for low plex chemistry) OK to release for publication, and not violate any NDA's with 10X?
Overall, this is a nice and informative paper. I am left with more concerns about the performance of the Prime assay itself than the combination of Prime and low plex chemistries.

Reviewer #3 (Comments to the Authors (Required)):

Williams-Katek et al. investigated the combination of a dual chemistry method for 10X Genomics Xenium spatial transcriptomic platform. They combined a high sensitivity custom V1 panel consisting of 480 genes of human lung tissues with a Prime panel of 5001 genes designed to encompass gene expression in a variety of human tissues on a single section. The final goal is to obtain spatial transcriptomics datasets with high per gene sensitivity to accurately identify cell types while also obtaining full transcriptome coverage. The authors show that combination of the two panels on one single section is technically possible and data from two consequent runs can be combined by using one single segmentation masks, allowing alignment of transcripts within the same cell coordinates. However, sensibility of the Prime panel is reduced in the dual run with regards to genes targeted by both chemistries.
This paper provides an interesting technical proof of concept. Combining V1 and Prime chemistries on one slide would enable higher sensitivity to detect cell types as well as offering discovery-oriented analyses. The major limitation is that the study does not comprehensively describe the true biological relevance of combining the two techniques that would justify the costs and time required to combine the two platforms on a single slide. For this, the study would greatly benefit from further bioinformatic analyses that demonstrate a true biological benefit.
Major comments:
1. The authors need to show whether cell populations and cell state can be identified with greater resolution comparing the dual run to solo runs, reaching beyond quality control parameters shown. Specifically:
a. Does the dual approach lead to greater and finer discrimination/annotation of cell types?
b. Does the dual approach allow to discriminate cell states within a single cell type?
c. It would be important to perform clustering and cell annotation based on the V1 panel in the dual samples and then overlay genes identified by the Prime panel to capture differences across cell types.
2. Figure 2b needs to be explained better. My understanding is that the dual run retains more cells after filtering, compared to V1 or Prime only runs.
a. The "unfiltered" sample is the dual run? This is not clear from the figure. I think it would help if also pre-filtering cell counts for V1 and Prime only are shown in the same figure and clearly describe that differences between V1 and Prime only are also expected.
b. In the text:
"these cutoffs were used to filter the 1,081,011 cells using transcripts only from the V1 panel, transcripts only from the Prime panel, and transcripts from the combined dataset".
It is not clear which group the 1,081,011 cells come from (presumably the Dual run) but it needs to be clear.
c. this sentence in the text is not associated to any data:
"Of the four filter parameters, we found that n_counts had the greatest impact on cell retention"
Can this be explained, as implication of this on filtering is not clear?
3. Figure 2e: the authors claim that there is an obvious imbalance in the panels with 538 secreted genes profiled with the Prime chemistry and only 59 profiled on V1. This imbalance is likely mostly driven by the abundance of genes within each panel as the V1 panel was designed specifically for use with human lung tissues and limited to a total of 480 genes. The density plot, despite showing a minimal increase in secreted genes per cell, remains of limited relevance. It would be more informative and impactful if the expression of these 538 secreted genes from the Prime panel is assessed across a set of different clusters as identified by analyses from the V1 panel.
4. Figure 3 is not clear. Panel a is of difficult interpretation and labels are missing in the alluvial plot in panel b, making it difficult

to compare conditions. I think this figure would benefit from different and more quantitative representations.
a. I suggest that other quantification analyses or representations are used to show concordance of clustering across protocols in panel b. From the alluvial plot is difficult to understand the quantitative difference between conditions.
b. To understand the impact and advantage of using the combined protocol, it is important to show cluster annotations and main marker genes identified per each cluster across conditions. This would allow to understand how the dual run benefits cluster annotation and cell identification.
c. Show the number of cells retained in each cluster by comparing Dual, V1 and Prime only.
d. Is cluster annotation cleaner in the dual run compared to V1 and Prime?
Minor comments:
1. TMA is not spelled out in the main text in results section.
2. Panels b to e in figure 1 are difficult to follow. I would suggest putting plots in panel b in row rather then column; panel c below panel b; panel d in row instead of column; panel e below panel d.
3. Change colors of V1 and Prime in panel e to be consistent with other panels (change to pink and light blue respectively. Maintain same color codes in the supplementary figures: S3 and S4).
4. Fig. S6. Can this be put in a graph, comparing correlation between solo run and dual run by chemistry? (it is very difficult to follow the corresponding text in the manuscript). text description does not immediately reflect the data reported in the table, where only raw counts are reported and not percentages. It would be useful to report these in a graph if possible, for ease of interpretation to the reader.

To the editor and reviewers:

We want to express our gratitude to all three reviewers for their thoughtful and thorough assessment of our submitted manuscript. We appreciate all of the feedback received surrounding this work and believe that the feedback has helped refine the paper for improved clarity of interpretation. As appropriately mentioned by Reviewer 1, spatial transcriptomics is a rapidly evolving field. We are incredibly excited to share this method with Xenium users who are in need of a combined high/low plex solution!

We have high hopes that the field will fill in certain gaps (established by this study) as technology advances, and we were curious as to whether or not this dual run approach was feasible at all. As such, we chose to run a TMA with samples that were already at our disposal, but our ability to increase our sample size is low without a significant cost burden. Unfortunately, we do not have readily-available access to sample types of diverse backgrounds that would be needed to fully validate this approach.

Additionally, almost all of the reviewers commented that the biological discoveries are limited by this fact. However, biological insight was not the goal of releasing this paper, and though we fully agree that concepts like cell type annotation would be a good clustering evaluatory metric, we unfortunately do not have the bandwidth at this moment in time to invest the energy into something as manual as cell type annotation. We simply wished to prove that it was technically possible to do a combined run with minimal effect on overall RNA quality, share our protocols, and release our lung benchmarking with the greater genomics community. We wanted to provide a current solution to encourage others to push the boundaries of presently available systems.

Despite the biological limitations of our dataset, in response to review comments, we have made technical revisions. These include figure rearrangements and removal of Figure 3 entirely. The secretome analysis has been exchanged for what we hope will be a more easily-interpreted ligand-receptor analysis. Some supplemental figures and tables have been expanded. The manuscript text has also been edited overall for improved explanations and clarity, focused on areas of confusion flagged by the reviewers. We thank the reviewers for their time and continued analysis of this manuscript!

Reviewer #1 (Comments to the Authors):
This manuscript presents a technically well-executed dual-chemistry spatial transcriptomics approach that combines Xenium V1 and Prime panels on the same tissue section. The benchmarking is thorough and the effort to bridge sensitivity and plex is appreciated. However, I have several concerns regarding the practical utility, scalability, and biological added value of this approach, especially in a field that is rapidly evolving toward higher plex and improved sensitivity within single assays.

We sincerely thank Reviewer #1 for their time and assessment of our work!

My concerns are:
Scalability: The current workflow requires sequential runs and dual chemistry preparation on the same slide. It is unclear how this approach could be scaled to larger studies or cohorts, particularly in high-throughput settings.
Cost: The method requires both V1 and Prime reagent kits and multiple instrument runs. This represents a substantial increase in cost per sample, which may limit adoption. A more quantitative discussion of cost vs. benefit would be valuable.

We appreciate the reviewer's comments and completely agree that the reagent price of the dual run approach could be cost prohibitive. We combined our scalability discussion with the purchase information in "Discussion" paragraph 2. While we have not formally tested the approach, we strongly believe that the suggested imputation method on a smaller subset of samples could be a promising way to alleviate the need for every slide to run the dual chemistry. This is especially true with the use of tissue microarrays, which can be designed to encompass and represent the variables within a larger study cohort.

Compatibility with protein detection It would be important to clarify whether this dual workflow is compatible with protein co-detection, which is increasingly a key feature of spatial platforms.

We thank the reviewer for this insightful thought! While not performed on this slide, we predict that the dual approach will be compatible with the protein co-detection currently available on the Xenium platform. We have added this information in "Discussion" paragraph 3. Since the protein tags are detected during the final phase of imaging cycles on the instrument, that decoding is kept separate from the transcript detection. On this slide, we utilized the cell morphology stains to enable cell segmentation. These stains are compatible with the protein panels and imaged/detected in the same manner. As such, it is expected that protein co-detection can be enabled with the dual chemistry approach.

Limited biological validation: The method is only demonstrated in one tissue type (human lung). It is unclear whether the same performance and trade-offs would hold across other tissues or disease contexts.

We completely agree that this is a limitation of our current manuscript. Unfortunately, it is simply out of the scope of our lab currently to validate a variety of tissues. Our lab is primarily human lung biology focused, so we have access to

these samples. However, other sample types are not readily available to us. As mentioned, this was also intended to be a small technical proof-of-concept manuscript, because we were unsure if the chemistry would be compatible at all. We would like the reviewer to know that these lung slides are part of a much larger unpublished dataset, so these samples will continue to be investigated for deeper biological insights, including cell-type annotations. However, that information simply is not available to be publicized at this time and it does not make sense to perform separate biological analyses on these slides outside of the context of the larger study. We thank the reviewer for their assessment and we are looking forward to what else may be discovered in the future.

Computational and technical overhead: The approach introduces additional steps (image registration, segmentation alignment, dataset integration), which increase computational burden and potential sources of variability. A discussion of robustness and reproducibility of these steps would strengthen the manuscript.

Additional text information has been added to “Results” paragraph 5. Information and software citations have been added to the “Methods” section “Cell mask transformation”. All software used is available through and validated by 10X Genomics. As such, we expect the results to be reproducible between slides and sample types, depending primarily on the initial DAPI image registration performed in the Xenium Explorer.

Unclear biological added value: While the technical integration is convincing, the biological advantage remains somewhat underdeveloped. The manuscript would benefit from a clearer demonstration of analyses or insights that are uniquely enabled by the dual approach and could not be achieved with existing high-plex or high-sensitivity methods alone.

We completely understand and agree with the reviewer that deeper insights would be ideal. As addressed above in the limited biological validation section, the lab is currently limited on the resources that can be dedicated to this proof-of-concept project at this time. In an effort to provide a more easily digestible example of potential analyses enabled by the dual chemistry approach, we have exchanged our original secretome analysis for a ligand-receptor pair analysis. This can be found in the final “Results” paragraph, Figure 2e, and Table S9. We believe this more accurately captures insights that can be gleaned from the additional panel without sacrificing V1 specificity.

In summary, this is a careful and well-benchmarked technical study. However, given the increased cost, complexity, and unclear scalability, the practical impact of this approach remains uncertain in the context of rapidly advancing spatial transcriptomics technologies. While the technical integration is convincing, the biological advantage is insufficiently demonstrated. It is not entirely clear what new biological insights or discoveries are uniquely enabled by the dual approach, beyond what could be achieved with a well-designed targeted panel or a high-plex panel alone. The examples provided are illustrative but do not establish a compelling use case where dual chemistry is necessary rather than incremental. The authors should more clearly

articulate and substantiate the unique biological value of this approach, and make a stronger case for why the added complexity is justified over existing alternatives.

We thank the reviewer for their thorough and thoughtful assessment of this manuscript.

Reviewer #2 (Comments to the Authors):

Summary:

The authors present a clever method to combine the high-sensitivity Xenium assay with high-breadth the Xenium assay, overcoming some of the trade-offs between targeted and discovery-based assays. This paper is very thorough and overall convinces me that the drawbacks in sensitivity associated with combining assays are relatively minimal, so users who cannot afford to trade off these elements should consider the approach. However, there are a few missing pieces to this manuscript that need to be addressed before recommending for publication.

We sincerely thank Reviewer #2 for their time and assessment of our work!

1. Aside from concordance between the solo vs. dual approach in transcript detection sensitivity (e.g. counts), it is important to consider whether localization of gene expression is broadly in agreement between the solo vs. dual assays. The authors do not show any images that represent transcript localization between the solo vs. dual approach. The authors visually compare the Prime vs. V1 results (dual vs. dual) across a few relatively small fields of view across 10 genes, which suggests a drastic drop in sensitivity in the Prime assay. The reviewer suggests that the authors show solo vs. dual, and tissue- or slide-level figures across more than just 10 genes to represent the level of agreement in transcript localization. This would be more interpretable than the clustering UMAP results that are not spatially localized as shown in Figure 3. An additional/alternative showcase could be to display the spatial representation of UMAP clusters on the dual vs. solo slides.

We thank the reviewer for their comments and suggestions! We have expanded Supplemental Figure 6 (formerly Fig. S5) to include the same field of views showing V1 solo and Prime solo detection of the 10 genes. We selected these genes to represent a range of cell lineages. While we agree that it would be ideal to show additional genes within these views, we believe that it makes the images too visually complex to interpret quickly (overlapping dots with too many colors). This is why we chose to rely more heavily on correlation scores between slides/samples and then supplemented with the visualization. At larger fields of view, the Xenium Explorer will also begin to downsample the number of visual transcripts, which we felt was not conducive to the point of supporting similar localization of individual gene transcripts. We fully recognize that this is an important comparison to make between all four slides, which is why we provide all raw data through the associated GEO accession (GSE315411). We would highly encourage anyone interested in attempting this chemistry to download the data and visualize the comparison across any number of available genes.

2. While the Prime assay has 40-60% reduced sensitivity on a per-gene basis in comparison to the low plex assay, it is very surprising that the authors are seeing a drop in total detected transcripts in the Prime assay (across 5,000 genes) compared to the V1 assay. This brings into question either the Prime assay execution, tissue quality, or relevance of the 5K panel to lung tissue. Can the authors comment on this? Things to consider are how old the archived FFPE

block is (this is not stated), what its DV200 score is, and how many genes from the 5K panel are expected to be relevant to the tissue type.

We thank the reviewer for their thoughtful comments for clarity on the source of the sensitivity drop in prime! We would like to first acknowledge that this is a phenomenon that our team has observed across 50+ unique hLung samples run on both Prime and V1 chemistry since the introduction of the Prime chemistry. We cannot definitively say that this would be the same across all tissue types, but we anticipate it to be the nature of the drop from 8 individual probesets per gene on the V1 panels to 3 probesets per gene on the Prime panels. These samples have been over a wide range of FFPE block ages. We have found the Xenium system to be one of the most robust spatial transcriptomic systems with regards to RNA quality. Due to the fact that these samples are of a more precious nature, we did not perform DV200 testing prior to running. However, we have added information regarding the age of the blocks into the "Methods" section "Sample Selection". These samples are part of a larger unpublished dataset and as such, cannot have full metadata released at this time.

We would also like to thank the reviewer for inquiring about the lung tissue compatibility with the 5K targets. It brings up an excellent point that the Prime panel is built to be universally applicable, and therefore cannot be considered to perform on the same level as tissue-specific panels. To this end, we have added some information about the Prime panel relevance to lung tissue in "Discussion" paragraph 1. We found that 392 genes are expected to be explicitly relevant to human lung tissue. However, many of the hallmark and high expressing genes are not present on the lung panel. This can partially explain why the total transcript detection is lower in Prime, despite the higher number of gene targets. We also believe that probe-probe interactions could be a source of the slight dip in performance of Prime on the dual slide which is discussed below in point 3.

3. Can the authors comment on compatibility of the 5K and low plex probes themselves? For example, is there cross-talk or complementary sequences in the barcode regions of the probes that could cause the Prime and low plex probes to spuriously bind to each other and amplify?

We appreciate this insightful idea from the reviewer! We had done some preliminary looking into the probe sequences, but decided that a more in depth analysis was needed. To investigate, we performed a BLAST analysis by querying probe sequences from the custom V1 panel against a custom database of the 5K probe sequences. We filtered the resulting hits based on the number of aligned nucleotides (Alignment Length ≧25), the similarity to the matched sequence (Percent Identity ≧90), and the possibility that the match could occur by random chance (Evalue ≤0.05) to identify gene probes that were most likely to be affected. We found a total of 142 genes had a high potential for probe binding between our custom V1 and the Prime panel, mostly between a probe designed for the same gene on the opposing panel (table can be found at the end of this PDF). Based on the fact that these binding events would not be anchored to the tissue, we expect

that the subsequent wash steps would remove them before they have the chance to amplify. However, sensitivity could still be affected by resulting in a lower concentration of probes reaching their appropriate targets within the tissue. Unfortunately, the Xenium instrument does not provide probe-level metrics of detection. All probes for a single gene target are decoded as just the gene name, leaving no way for us to validate that the probe hits on BLAST were actually being detected at lower levels. As we are unable to close the loop on this analysis, we feel it is best to keep this idea in the manuscript "Results" paragraph 3, but keep our main analysis focused on the correlative performance of all 239 overlapping genes. We do agree with the reviewer that the probe sequences could provide helpful information and, as such, have made the FASTA files available on GEO for both the human Prime panel and our custom human lung V1 panel.

4. Can the authors comment on how false positive rates are affected by the dual run?

Information on the false positive rates for all four slides has been added to "Results" paragraph 3. Higher false positive rates are present on the V1 dual detection than any others. The V1 rates overall are also higher than the Prime rates. We believe this may be further indication that the higher number of probesets present in V1 panels could be responsible for the increased binding, both to their expected and spurious targets.

5. Is the information from 10X for combining 5K + low plex assays (particularly the oligo sequence for low plex chemistry) OK to release for publication, and not violate any NDA's with 10X?

We thank the reviewer for this question. While the inclusion of this oligo does not violate any NDAs, upon review, we decided to remove this section of the Discussion since we had not tested it in our own system. We also believe that it would not have enabled too substantial of a reagent price reduction in light of the overall cost of the process. It would only remove the need for the full V1 sample prep reagents (list price $1350) and would still require the purchase of a custom panel, V1 decoding supplies, and all Prime reagents. We believe that the imputation suggestion in "Discussion" paragraph 2 is a more reasonable approach to limiting the cost of applying this technique to large datasets.

Overall, this is a nice and informative paper. I am left with more concerns about the performance of the Prime assay itself than the combination of Prime and low plex chemistries.

We thank the reviewer for their assessment! The data from the Prime slides falls within the expected specifications from 10X Genomics. We agree the sensitivity of this assay is considerably lower than the V1 assay.

Reviewer #3 (Comments to the Authors):
Williams-Katek et al. investigated the combination of a dual chemistry method for 10X Genomics Xenium spatial transcriptomic platform. They combined a high sensitivity custom V1 panel consisting of 480 genes of human lung tissues with a Prime panel of 5001 genes designed to encompass gene expression in a variety of human tissues on a single section. The final goal is to obtain spatial transcriptomics datasets with high per gene sensitivity to accurately identify cell types while also obtaining full transcriptome coverage. The authors show that combination of the two panels on one single section is technically possible and data from two consequent runs can be combined by using one single segmentation masks, allowing alignment of transcripts within the same cell coordinates. However, sensibility of the Prime panel is reduced in the dual run with regards to genes targeted by both chemistries.
This paper provides an interesting technical proof of concept. Combining V1 and Prime chemistries on one slide would enable higher sensitivity to detect cell types as well as offering discovery-oriented analyses. The major limitation is that the study does not comprehensively describe the true biological relevance of combining the two techniques that would justify the costs and time required to combine the two platforms on a single slide. For this, the study would greatly benefit from further bioinformatic analyses that demonstrate a true biological benefit.

We sincerely thank Reviewer #3 for their time and assessment of our work!

Major comments:
1. The authors need to show whether cell populations and cell state can be identified with greater resolution comparing the dual run to solo runs, reaching beyond quality control parameters shown. Specifically:
a. Does the dual approach lead to greater and finer discrimination/annotation of cell types?
b. Does the dual approach allow to discriminate cell states within a single cell type?
c. It would be important to perform clustering and cell annotation based on the V1 panel in the dual samples and then overlay genes identified by the Prime panel to capture differences across cell types.

We appreciate this suggestion from the reviewer and agree that the use of cell-type annotations would be ideal for showing the benefits of the dual approach for deep biological significance. Unfortunately, this level of data analysis is not feasible with currently available resources in the lab. We would like the reviewer to know that these lung slides are part of a much larger unpublished dataset. As a result, these samples will continue to be investigated for deeper biological insights, including cell-type and state annotations. However, that information simply is not available to be publicized at this time and it does not make sense to perform separate biological analyses on these slides outside of the context of the larger study. We thank the reviewer for their assessment and we are looking forward to what else may be discovered in the future.

2. Figure 2b needs to be explained better. My understanding is that the dual run retains more cells after filtering, compared to V1 or Prime only runs.

a. The "unfiltered" sample is the dual run? This is not clear from the figure. I think it would help if also pre-filtering cell counts for V1 and Prime only are shown in the same figure and clearly describe that differences between V1 and Prime only are also expected.

We thank the reviewer for this feedback and have worked to make this section more easily comprehensible. We have added an additional supplementary figure (Figure S5) which includes a bar chart of the pre- and post-filtering cell counts of the two solo slides. We believe this will be helpful in setting the expectation for what amount of cell loss is typical when quality control filtering Xenium hLung spatial data for downstream analysis. We have also translated the raw numbers available in the chart into cell retention percentages in the text ("Results" paragraph 5) to provide an immediately available comparison with the dual slide percentages. We see that the patterns of cell loss are consistent in both the solo and dual chemistry, with the combined gene set (5242 total unique genes) allowing the most cells to have enough data available to pass our filtering metrics.

Solo V1 cell retention = 60.3%
Dual V1 cell retention = 59.6%
Solo Prime cell retention = 51.4%
Dual Prime cell retention = 47.2%
Dual Combined cell retention = 75.5%

b. In the text:
"these cutoffs were used to filter the 1,081,011 cells using transcripts only from the V1 panel, transcripts only from the Prime panel, and transcripts from the combined dataset".
It is not clear which group the 1,081,011 cells come from (presumably the Dual run) but it needs to be clear.

To improve clarity, the text associated with Figure 2b has been updated to include "cells segmented on the dual slide" with regards to the "unfiltered" sample in the graph ("Results" paragraph 5).

c. this sentence in the text is not associated to any data:
"Of the four filter parameters, we found that n_counts had the greatest impact on cell retention"
Can this be explained, as implication of this on filtering is not clear?

We thank the reviewer for highlighting this sentence for examination. The reviewer is correct that there is no quantitative data associated with this statement. It was simply a pattern observed during the filtering process. We believe that it suggests that the transfer of the cell segmentation masks was not impacting cell quality metrics and that the gene panel chemistry used to filter the number of transcripts was more important. However, since we did not make a formal comparison, we have removed this sentence from the manuscript.

3. Figure 2e: the authors claim that there is an obvious imbalance in the panels with 538 secreted genes profiled with the Prime chemistry and only 59 profiled on V1. This imbalance is likely mostly driven by the abundance of genes within each panel as the V1 panel was designed

specifically for use with human lung tissues and limited to a total of 480 genes. The density plot, despite showing a minimal increase in secreted genes per cell, remains of limited relevance. It would be more informative and impactful if the expression of these 538 secreted genes from the Prime panel is assessed across a set of different clusters as identified by analyses from the V1 panel.

We sincerely appreciate the reviewer's thoughts and suggestions regarding our secretome analysis. Upon reassessment of the results, we agree with the reviewer that this was not the ideal method to showcase a biological use case of the dual chemistry. As mentioned in point 1 above, we did not perform a full cell-type annotation of this dataset so we did not feel that we could appropriately improve this analysis. We decided to transition to a ligand-receptor (LR) analysis with lineage-level cell annotation (epithelial, endothelial, mesenchymal, immune). We focused on epithelial ligands and mesenchymal receptors within neighboring cells close enough to physically interact (between k=1 nearest neighboring cells within a Euclidean distance of ≤ 10μm) based on a human LR pair database from CellTalkDB. We recognize that other more distant ligand-receptor interactions can occur, but we felt that would convolute the data interpretation. We performed the analysis independently for each panel and identified interactions detected in both panels as overlapping hits. Within the dual slide cells, 27 overlapping LR pairs were identified as interacting within our samples in both the V1 and Prime chemistries. 78 unique LR pairs were identified in the V1 chemistry and 635 unique LR pairs were identified in the Prime chemistry. We believe that this is a more concrete example of an analysis made possible by the dual chemistry. While the sensitivity of the V1 panel is ideal for very targeted investigations, the addition of the Prime panel enables more discovery-based analyses (such as LR) within the same samples.

"Methods" sections "Clustering and lineage annotation", "Cellular proximity", and "Ligand-receptor analysis" have been added to explain the process.
Figure 2e has been updated with a Venn diagram demonstrating the detected LR pair distributions across chemistries on the dual slide.
Supplementary Table S8 has been updated to reflect the LR pairs queried in this analysis as opposed to the original table reflecting the secretory genes.

4. Figure 3 is not clear. Panel a is of difficult interpretation and labels are missing in the alluvial plot in panel b, making it difficult to compare conditions. I think this figure would benefit from different and more quantitative representations.
a. I suggest that other quantification analyses or representations are used to show concordance of clustering across protocols in panel b. From the alluvial plot is difficult to understand the quantitative difference between conditions.
b. To understand the impact and advantage of using the combined protocol, it is important to show cluster annotations and main marker genes identified per each cluster across conditions.

This would allow to understand how the dual run benefits cluster annotation and cell identification.
c. Show the number of cells retained in each cluster by comparing Dual, V1 and Prime only.
d. Is cluster annotation cleaner in the dual run compared to V1 and Prime?

We completely agree with the reviewer's assessment that Figure 3 is difficult to interpret. After multiple iterations during the revision process, we have decided to remove Figure 3 in its entirety. We tested other visualizations of the data, but it simply does not communicate added biological information without the clusters being annotated as cell types. In line with comments above to Point 1 as well as to other reviewers, we are unable to provide that depth of information at this time. Figure 3 largely showed that clustering was similar enough, but we do not believe it was compelling enough to keep in the manuscript as a presentation. The other goal of the figure was to demonstrate that discovery was improved by the combined resolution of the dual chemistry, as we had more cells overall that passed quality filters to be clustered shown in the alluvial plot. Cells that were otherwise placed in the "NA" category using only one panel or the other. We believe that this point is already well-made by the pre- and post- filtering bar charts of Figure 2b and Figure S5, making Figure 3b largely redundant in the context of the full manuscript. Overall, we believe the removal of Figure 3 will remove a large source of confusion from the paper, since we do not currently have the level of granularity that such an analysis requires.

Minor comments:
1. TMA is not spelled out in the main text in results section.

We thank the reviewer for catching this oversight! The spelling "tissue microarray (TMA)" has been added to the first sentence in "Results" paragraph 1. The acronym "(TMA)" has also been added to the "Abstract".

2. Panels b to e in figure 1 are difficult to follow. I would suggest putting plots in panel b in row rather then column; panel c below panel b; panel d in row instead of column; panel e below panel d.

We agree with the reviewer that the original layout was suboptimal. Figure 1 has been reorganized for easier flow between panels. Figure 1b, 1c, 1d, and 1e all now align from left to right by adjusting 1c and 1e into a vertical format.

3. Change colors of V1 and Prime in panel e to be consistent with other panels (change to pink and light blue respectively. Maintain same color codes in the supplementary figures: S3 and S4).

We appreciate the reviewer's suggestion to align color schemes in these panels and supplementary figures, but did not make this adjustment. The fact that the colors do not match between these specific panels in Figure 1 was an intentional decision. The early panels, 1b and 1d, are depicting gene panel-level metrics across both runs. Panel 1e is depicting an instrument run-level decoding metric (V1 versus Prime). The colors in panel 1e are then continued in figures S3 and S4 as those contain instrument run-level decoding metrics broken down by sample.

4. Fig. S6. Can this be put in a graph, comparing correlation between solo run and dual run by chemistry? (it is very difficult to follow the corresponding text in the manuscript). text description does not immediately reflect the data reported in the table, where only raw counts are reported and not percentages. It would be useful to report these in a graph if possible, for ease of interpretation to the reader.

We thank the reviewer for this comment, but are a bit unsure of how to address this request. We are unaware of another graph to more appropriately show correlation patterns between raw transcript counts. Figure S6 is a correlation scatterplot. "Percentages" are not referred to in the figure, figure legend, nor the associated manuscript text. Nevertheless, we have reviewed all figures with an eye on ensuring that figures and legends are matched.

Please note that an additional supplementary figure has been added, so the referred-to original "Fig. S6" is now Figure S7 in the revised manuscript.

Custom BLAST results querying each custom V1 probe sequence against all probes in the Prime panel												
Query	Subject	Percent Identity	Alignment Length	Mismatches	Gaps	Query Start Match	Query End Match	Subject Start Match	Subject End Match	Evalue	Bitscore	Source Panel ID of Query
ACAA2	ACAA2	100	32	0	0	5	36	1	32	4.76E-12	60.2	43R3RN
ADAMTS1	ADAMTS1	100	32	0	0	1	32	1	32	4.76E-12	60.2	6ZAAWG
ADGRL4	ADGRL4	100	32	0	0	4	35	1	32	4.76E-12	60.2	6ZAAWG
AGR3	AGR3	100	31	0	0	10	40	1	31	1.71E-11	58.4	6ZAAWG
APLNR	APLNR	100	30	0	0	11	40	1	30	6.15E-11	56.5	43R3RN
ARF6	ARF6	100	30	0	0	11	40	1	30	6.15E-11	56.5	43R3RN
ASPN	ASPN	100	28	0	0	13	40	1	28	7.96E-10	52.8	6ZAAWG
ATG7	ATG7	100	32	0	0	4	35	1	32	4.76E-12	60.2	6ZAAWG
BANK1	BANK1	100	32	0	0	6	37	1	32	4.76E-12	60.2	6ZAAWG
BAX	BAX	100	25	0	0	1	25	8	32	3.70E-08	47.3	6ZAAWG
BAX	BAX	100	31	0	0	1	31	2	32	1.71E-11	58.4	6ZAAWG
BCL2	BCL2	100	32	0	0	5	36	1	32	4.76E-12	60.2	6ZAAWG
BCL2L11	BCL2L11	100	32	0	0	6	37	1	32	4.76E-12	60.2	6ZAAWG
BMP4	BMP4	100	32	0	0	4	35	1	32	4.76E-12	60.2	6ZAAWG
BMP4	BMP4	100	26	0	0	1	26	7	32	1.03E-08	49.1	6ZAAWG
BMP4	BMP4	100	32	0	0	6	37	1	32	4.76E-12	60.2	6ZAAWG
BMPR2	BMPR2	100	32	0	0	5	36	1	32	4.76E-12	60.2	6ZAAWG
CCL18	CCL18	100	32	0	0	1	32	1	32	4.76E-12	60.2	43R3RN
CCN2	CCN2	100	32	0	0	4	35	1	32	4.76E-12	60.2	6ZAAWG
CD14	CD14	100	31	0	0	1	31	2	32	1.71E-11	58.4	6ZAAWG
CD14	CD14	100	31	0	0	10	40	1	31	1.71E-11	58.4	6ZAAWG
CD151	CD151	100	29	0	0	1	29	4	32	2.21E-10	54.7	6ZAAWG
CD163	CD163	100	32	0	0	4	35	1	32	4.76E-12	60.2	6ZAAWG
CD19	CD19	100	32	0	0	4	35	1	32	4.76E-12	60.2	6ZAAWG
CD19	CD19	100	32	0	0	6	37	1	32	4.76E-12	60.2	6ZAAWG
CD1C	CD1C	100	28	0	0	1	28	5	32	7.96E-10	52.8	6ZAAWG
CD1C	CD1C	100	32	0	0	5	36	1	32	4.76E-12	60.2	6ZAAWG
CD1E	CD1E	100	27	0	0	1	27	6	32	2.86E-09	51	6ZAAWG
CD1E	CD1E	100	32	0	0	4	35	1	32	4.76E-12	60.2	6ZAAWG
CD247	CD247	100	32	0	0	1	32	1	32	4.76E-12	60.2	6ZAAWG
CD27	CD27	100	32	0	0	5	36	1	32	4.76E-12	60.2	6ZAAWG
CD27	CD27	100	31	0	0	1	31	2	32	1.71E-11	58.4	6ZAAWG
CD34	CD34	100	28	0	0	1	28	5	32	7.96E-10	52.8	6ZAAWG
CD34	CD34	100	32	0	0	6	37	1	32	4.76E-12	60.2	6ZAAWG
CD3E	CD3E	100	32	0	0	1	32	1	32	4.76E-12	60.2	6ZAAWG
CD3G	CD3G	100	30	0	0	11	40	1	30	6.15E-11	56.5	6ZAAWG
CD4	CD4	100	32	0	0	2	33	1	32	4.76E-12	60.2	6ZAAWG
CD4	CD4	100	29	0	0	1	29	4	32	2.21E-10	54.7	6ZAAWG
CD4	CD4	100	32	0	0	4	35	1	32	4.76E-12	60.2	6ZAAWG
CD44	CD44	100	29	0	0	12	40	1	29	2.21E-10	54.7	6ZAAWG
CD47	CD47	100	29	0	0	1	29	4	32	2.21E-10	54.7	6ZAAWG
CD79A	CD79A	100	31	0	0	1	31	2	32	1.57E-11	58.4	6ZAAWG
CD79A	CD79A	100	32	0	0	2	33	1	32	4.76E-12	60.2	6ZAAWG
CD79B	CD79B	100	27	0	0	14	40	1	27	2.86E-09	51	6ZAAWG
CD8A	CD8A	100	32	0	0	5	36	1	32	4.76E-12	60.2	6ZAAWG
CD8B	CD8B	100	26	0	0	1	26	7	32	1.03E-08	49.1	6ZAAWG
CD8B	CD8B	100	30	0	0	11	40	1	30	6.15E-11	56.5	6ZAAWG
CES1	CES1	100	32	0	0	7	38	1	32	4.76E-12	60.2	6ZAAWG
CFTR	CFTR	100	32	0	0	2	33	1	32	4.76E-12	60.2	6ZAAWG
CH25H	CH25H	100	25	0	0	1	25	8	32	3.70E-08	47.3	43R3RN
CH25H	CH25H	100	28	0	0	1	28	5	32	7.96E-10	52.8	43R3RN
CHIT1	CHIT1	100	32	0	0	1	32	1	32	4.76E-12	60.2	6ZAAWG
CHIT1	CHIT1	100	30	0	0	1	30	3	32	6.15E-11	56.5	6ZAAWG
CLDN5	CLDN5	100	30	0	0	11	40	1	30	6.15E-11	56.5	6ZAAWG
COL4A1	COL4A1	100	32	0	0	4	35	1	32	4.76E-12	60.2	6ZAAWG
COL4A3	COL4A3	100	30	0	0	11	40	1	30	6.15E-11	56.5	43R3RN
CTHRC1	CTHRC1	100	32	0	0	7	38	1	32	4.76E-12	60.2	6ZAAWG
CXCL13	CXCL13	100	31	0	0	10	40	1	31	1.71E-11	58.4	6ZAAWG
CXCL6	CXCL6	100	32	0	0	5	36	1	32	4.76E-12	60.2	6ZAAWG
CXCR4	CXCR4	100	32	0	0	6	37	1	32	4.76E-12	60.2	6ZAAWG
DERL3	DERL3	100	29	0	0	12	40	1	29	2.21E-10	54.7	6ZAAWG
DNAH12	DNAH12	100	28	0	0	13	40	1	28	7.96E-10	52.8	6ZAAWG
ELANE	ELANE	100	32	0	0	7	38	1	32	4.38E-12	60.2	43R3RN
ELANE	ELANE	100	32	0	0	8	39	1	32	4.76E-12	60.2	43R3RN
ELANE	ELANE	100	31	0	0	1	31	2	32	1.71E-11	58.4	43R3RN
EPAS1	EPAS1	100	30	0	0	1	30	3	32	6.15E-11	56.5	6ZAAWG
EPAS1	EPAS1	100	32	0	0	9	40	1	32	4.76E-12	60.2	6ZAAWG
EREG	EREG	100	29	0	0	1	29	4	32	2.21E-10	54.7	6ZAAWG
EREG	CC2D1A	92.308	26	1	1	2	27	27	3	8.01E-05	36.2	6ZAAWG
FAP	FAP	100	32	0	0	5	36	1	32	4.76E-12	60.2	6ZAAWG
FAP	FAP	100	32	0	0	8	39	1	32	4.76E-12	60.2	6ZAAWG

FASN	FASN	100	32	0	0	2	33	1	32	4.76E-12	60.2	6ZAAWG
FCER1A	FCER1A	100	27	0	0	14	40	1	27	2.86E-09	51	6ZAAWG
FCGBP	FCGBP	100	28	0	0	13	40	1	28	7.96E-10	52.8	6ZAAWG
FCGBP	FCGBP	100	32	0	0	1	32	1	32	4.76E-12	60.2	6ZAAWG
FCGR3A	FCGR3A	100	27	0	0	1	27	6	32	2.86E-09	51	6ZAAWG
FGF7	FGF7	100	25	0	0	16	40	1	25	3.70E-08	47.3	43R3RN
FGFR4	FGFR4	100	32	0	0	5	36	1	32	4.76E-12	60.2	6ZAAWG
FKBP11	FKBP11	100	29	0	0	1	29	4	32	2.21E-10	54.7	6ZAAWG
FKBP11	FKBP11	100	26	0	0	1	26	7	32	9.47E-09	49.1	6ZAAWG
FOXJ1	FOXJ1	100	30	0	0	1	30	3	32	5.66E-11	56.5	6ZAAWG
FOXJ1	FOXJ1	100	29	0	0	12	40	1	29	2.21E-10	54.7	6ZAAWG
FSTL1	FSTL1	100	32	0	0	2	33	1	32	4.76E-12	60.2	6ZAAWG
GATA6	GATA6	100	30	0	0	1	30	3	32	6.15E-11	56.5	6ZAAWG
GATA6	GATA6	100	32	0	0	7	38	1	32	4.76E-12	60.2	6ZAAWG
GBP1	GBP1	100	32	0	0	8	39	1	32	4.76E-12	60.2	43R3RN
GBP1	GBP1	100	27	0	0	1	27	6	32	2.86E-09	51	43R3RN
GCLM	GCLM	100	32	0	0	5	36	1	32	4.76E-12	60.2	6ZAAWG
GDF15	GDF15	100	32	0	0	3	34	1	32	4.38E-12	60.2	6ZAAWG
GSR	GSR	100	32	0	0	4	35	1	32	4.76E-12	60.2	6ZAAWG
HDAC9	HDAC9	100	32	0	0	7	38	1	32	4.76E-12	60.2	6ZAAWG
HES1	HES1	100	31	0	0	10	40	1	31	1.71E-11	58.4	6ZAAWG
HEY1	HEY1	100	32	0	0	5	36	1	32	4.76E-12	60.2	6ZAAWG
HIF1A	HIF1A	100	31	0	0	1	31	2	32	1.71E-11	58.4	6ZAAWG
HMGA1	HMGA1	100	27	0	0	1	27	6	32	2.86E-09	51	6ZAAWG
HRAS	HRAS	100	32	0	0	6	37	1	32	4.38E-12	60.2	43R3RN
ICAM1	ICAM1	100	32	0	0	9	40	1	32	4.76E-12	60.2	6ZAAWG
ICAM1	ICAM1	100	30	0	0	11	40	1	30	6.15E-11	56.5	6ZAAWG
IDH1	IDH1	100	29	0	0	12	40	1	29	2.21E-10	54.7	6ZAAWG
IDH1	IDH1	100	32	0	0	2	33	1	32	4.76E-12	60.2	6ZAAWG
IFI16	IFI16	100	25	0	0	1	25	8	32	3.70E-08	47.3	43R3RN
IFT57	IFT57	100	32	0	0	4	35	1	32	4.76E-12	60.2	43R3RN
IFT57	IFT57	100	32	0	0	7	38	1	32	4.76E-12	60.2	43R3RN
IRF1	IRF1	100	29	0	0	1	29	4	32	2.21E-10	54.7	6ZAAWG
IRF1	IRF1	100	32	0	0	5	36	1	32	4.76E-12	60.2	6ZAAWG
IRF7	IRF7	100	32	0	0	4	35	1	32	4.38E-12	60.2	6ZAAWG
ITGA3	ITGA3	100	32	0	0	9	40	1	32	4.76E-12	60.2	43R3RN
ITGB6	ITGB6	100	30	0	0	1	30	3	32	6.15E-11	56.5	43R3RN
ITGB6	ITGB6	100	32	0	0	6	37	1	32	4.76E-12	60.2	43R3RN
KDR	KDR	100	32	0	0	8	39	1	32	4.76E-12	60.2	6ZAAWG
KIT	KIT	100	26	0	0	15	40	1	26	1.03E-08	49.1	6ZAAWG
KLRB1	KLRB1	100	32	0	0	4	35	1	32	4.76E-12	60.2	6ZAAWG
KLRC1	KLRC1	100	32	0	0	7	38	1	32	4.76E-12	60.2	6ZAAWG
KLRG1	KLRG1	100	27	0	0	14	40	1	27	2.86E-09	51	6ZAAWG
LCK	LCK	100	27	0	0	14	40	1	27	2.86E-09	51	6ZAAWG
LILRA4	LILRA4	100	32	0	0	7	38	1	32	4.76E-12	60.2	6ZAAWG
LILRA4	LILRA4	100	28	0	0	1	28	5	32	7.96E-10	52.8	6ZAAWG
LTBP2	LTBP2	100	32	0	0	8	39	1	32	4.76E-12	60.2	6ZAAWG
LTBP2	LTBP2	92	25	1	1	2	25	5	29	2.88E-04	34.4	6ZAAWG
LTBP2	LTBP2	100	32	0	0	6	37	1	32	4.76E-12	60.2	6ZAAWG
MAD1L1	MAD1L1	100	32	0	0	9	40	1	32	4.76E-12	60.2	6ZAAWG
MAD1L1	MAD1L1	100	32	0	0	7	38	1	32	4.76E-12	60.2	6ZAAWG
MARCO	MARCO	100	32	0	0	2	33	1	32	4.76E-12	60.2	6ZAAWG
MARCO	MARCO	100	31	0	0	10	40	1	31	1.71E-11	58.4	6ZAAWG
MLPH	MLPH	100	32	0	0	4	35	1	32	4.76E-12	60.2	6ZAAWG
MMP12	MMP12	100	26	0	0	15	40	1	26	1.03E-08	49.1	6ZAAWG
MMP12	MMP12	100	32	0	0	5	36	1	32	4.76E-12	60.2	6ZAAWG
MS4A1	MS4A1	100	31	0	0	1	31	2	32	1.71E-11	58.4	6ZAAWG
MS4A1	MS4A1	100	32	0	0	5	36	1	32	4.76E-12	60.2	6ZAAWG
MS4A1	MS4A1	100	28	0	0	13	40	1	28	7.96E-10	52.8	6ZAAWG
MSLN	MSLN	100	32	0	0	5	36	1	32	4.76E-12	60.2	6ZAAWG
MUC5B	MUC5B	100	32	0	0	8	39	1	32	4.76E-12	60.2	6ZAAWG
MYC	MYC	100	32	0	0	6	37	1	32	4.76E-12	60.2	6ZAAWG
NRP1	NRP1	100	32	0	0	8	39	1	32	4.76E-12	60.2	6ZAAWG
NTN4	NTN4	100	31	0	0	1	31	2	32	1.71E-11	58.4	6ZAAWG
NTN4	NTN4	100	25	0	0	1	25	8	32	3.70E-08	47.3	6ZAAWG
NUCB2	NUCB2	100	32	0	0	9	40	1	32	4.76E-12	60.2	6ZAAWG
PDIA4	PDIA4	100	32	0	0	4	35	1	32	4.76E-12	60.2	6ZAAWG
PDIA4	PDIA4	100	27	0	0	14	40	1	27	2.86E-09	51	6ZAAWG
PI16	PI16	100	32	0	0	4	35	1	32	4.76E-12	60.2	6ZAAWG
PIM2	PIM2	100	30	0	0	1	30	3	32	6.15E-11	56.5	6ZAAWG
PLVAP	PLVAP	100	30	0	0	1	30	3	32	6.15E-11	56.5	6ZAAWG
POSTN	POSTN	100	32	0	0	4	35	1	32	4.76E-12	60.2	6ZAAWG
POSTN	POSTN	100	32	0	0	6	37	1	32	4.76E-12	60.2	6ZAAWG

PPARG	PPARG	100	32	0	0	5	36	1	32	4.76E-12	60.2	6ZAAWG
PROM1	PROM1	100	32	0	0	5	36	1	32	4.76E-12	60.2	6ZX7JP
PTGS1	PTGS1	100	28	0	0	13	40	1	28	7.96E-10	52.8	6ZAAWG
PTPN6	PTPN6	100	32	0	0	5	36	1	32	4.76E-12	60.2	6ZX7JP
RNASEH2C	KAT5	100	32	0	0	8	39	32	1	4.76E-12	60.2	6ZX7JP
RSPO2	RSPO2	100	32	0	0	4	35	1	32	4.76E-12	60.2	6ZAAWG
RSPO2	RSPO2	100	32	0	0	1	32	1	32	4.76E-12	60.2	6ZAAWG
RSPO3	RSPO3	100	30	0	0	1	30	3	32	6.15E-11	56.5	6ZAAWG
RTKN2	RTKN2	100	32	0	0	5	36	1	32	4.76E-12	60.2	6ZAAWG
SELL	SELL	100	32	0	0	8	39	1	32	4.76E-12	60.2	6ZAAWG
SEMA3A	SEMA3A	100	32	0	0	5	36	1	32	4.76E-12	60.2	6ZAAWG
SFRP1	SFRP1	100	31	0	0	10	40	1	31	1.71E-11	58.4	6ZAAWG
SFRP1	SFRP1	100	28	0	0	13	40	1	28	7.96E-10	52.8	6ZAAWG
SLC25A4	SLC25A4	100	32	0	0	8	39	1	32	4.76E-12	60.2	6ZAAWG
SLC25A4	SLC25A4	100	26	0	0	1	26	7	32	1.03E-08	49.1	6ZAAWG
SLIT3	SLIT3	100	26	0	0	15	40	1	26	1.03E-08	49.1	6ZAAWG
SMPD3	SMPD3	100	26	0	0	1	26	7	32	1.03E-08	49.1	6ZAAWG
SMPD3	SMPD3	100	32	0	0	1	32	1	32	4.76E-12	60.2	6ZAAWG
SNAI1	SNAI1	100	32	0	0	7	38	1	32	4.76E-12	60.2	6ZAAWG
SNAI1	SNAI1	100	26	0	0	15	40	1	26	1.03E-08	49.1	6ZAAWG
SOX9	SOX9	100	32	0	0	5	36	1	32	4.76E-12	60.2	6ZAAWG
SPATS2L	SPATS2L	100	28	0	0	1	28	5	32	7.96E-10	52.8	6ZX7JP
SPATS2L	SPATS2L	100	32	0	0	5	36	1	32	4.76E-12	60.2	6ZX7JP
SPRY2	SPRY2	100	31	0	0	1	31	2	32	1.71E-11	58.4	6ZAAWG
SRSF1	SRSF1	100	26	0	0	15	40	1	26	1.03E-08	49.1	6ZX7JP
SRSF1	SRSF1	100	26	0	0	1	26	7	32	1.03E-08	49.1	6ZX7JP
STAT1	STAT1	100	32	0	0	3	34	1	32	4.76E-12	60.2	6ZAAWG
STAT6	STAT6	100	26	0	0	1	26	7	32	1.03E-08	49.1	6ZAAWG
SVEP1	SVEP1	100	26	0	0	1	26	7	32	1.03E-08	49.1	6ZAAWG
TBX4	TBX4	100	30	0	0	1	30	3	32	6.15E-11	56.5	6ZAAWG
TBX4	TBX4	100	32	0	0	5	36	1	32	4.76E-12	60.2	6ZAAWG
TCL1A	TCL1A	100	32	0	0	1	32	1	32	4.38E-12	60.2	6ZAAWG
TGFB1	TGFB1	100	31	0	0	1	31	2	32	1.71E-11	58.4	6ZAAWG
TGFB2	TGFB2	100	25	0	0	1	25	8	32	3.70E-08	47.3	6ZAAWG
TGFB2	TGFB2	100	29	0	0	12	40	1	29	2.21E-10	54.7	6ZAAWG
TNFRSF13C	TNFRSF13C	100	32	0	0	9	40	1	32	4.76E-12	60.2	6ZAAWG
TP53	TP53	100	32	0	0	1	32	1	32	4.76E-12	60.2	6ZAAWG
TREM2	TREM2	100	32	0	0	6	37	1	32	4.76E-12	60.2	6ZAAWG
TRIM47	TRIM47	100	27	0	0	12	38	1	27	2.63E-09	51	6ZX7JP
TRIM47	TRIM47	100	30	0	0	1	30	3	32	5.66E-11	56.5	6ZX7JP
TSPAN4	TSPAN4	100	32	0	0	2	33	1	32	4.76E-12	60.2	6ZX7JP
UNC119	UNC119	100	30	0	0	11	40	1	30	6.15E-11	56.5	6ZX7JP
UPK3B	UPK3B	100	30	0	0	11	40	1	30	6.15E-11	56.5	6ZAAWG
UPK3B	UPK3B	100	32	0	0	6	37	1	32	4.76E-12	60.2	6ZAAWG
UPK3B	UPK3B	100	27	0	0	12	38	1	27	2.63E-09	51	6ZAAWG
VEGFA	VEGFA	100	32	0	0	9	40	1	32	4.76E-12	60.2	6ZAAWG
WNT2	WNT2	100	32	0	0	8	39	1	32	4.76E-12	60.2	6ZAAWG
WNT3A	WNT3A	100	27	0	0	1	27	6	32	2.86E-09	51	6ZAAWG
WNT3A	WNT3A	100	28	0	0	1	28	5	32	7.96E-10	52.8	6ZAAWG
WNT5A	WNT5A	100	32	0	0	5	36	1	32	4.76E-12	60.2	6ZAAWG
WT1	WT1	100	32	0	0	5	36	1	32	4.76E-12	60.2	6ZAAWG
WWTR1	WWTR1	100	32	0	0	5	36	1	32	4.76E-12	60.2	6ZAAWG

June 30, 2026

RE: Life Science Alliance Manuscript #LSA-2026-03690R

Ms. Arianna L. Williams-Katek
Translational Genomics Research Institute
445 N 5th St
Phoenix, AZ 85004

Dear Dr. Williams-Katek,

Thank you for submitting your revised manuscript entitled "Fishing with Two Lines: A Hybrid Approach to Spatial Transcriptomic Discovery". We returned this work to all three reviewers, whose comments are below.

As you will see, reviewers agree that the proof-of-concept established by this manuscript is sufficiently supported by the data shown and presents a broadly important capability of the Xenium spatial transcriptomics platform. Reviewers 1 and 3 maintain that the addition of greater biological insight would enhance the overall interest in this work. We note the inability to secure additional human specimens to extend these observations and we agree that the current work will interest the field as a demonstration of feasibility. We would be happy to publish your paper in Life Science Alliance pending final revisions necessary to meet our formatting guidelines.

MANUSCRIPT ORGANIZATION AND FORMATTING:

To avoid unnecessary delays in the acceptance and publication of your paper, please read the following information carefully. Full guidelines are available on our Instructions for Authors page, https://www.life-science-alliance.org/authors

Submission of a paper that does not conform to Life Science Alliance guidelines will delay the acceptance of your manuscript.

-Please upload all figure files as individual ones, including the supplementary figure files; all figure legends should only appear in the main manuscript file.
-Please add the X and Bluesky handles of your host institute/organization, as well as your own, and/or one of the authors, in our system.
-It is recommended to exclude figures from the manuscript text and upload them separately.
-For publication, we require PowerPoint, TIFF, PDF, or EPS files. Please remove duplicated figures.
-Please add your main, supplementary figure, and table legends to the main manuscript text after the references section.
-Please add the Acknowledgements section to your manuscript text.
-Please add information on institutional approval to use human tissue to the Methods section "Sample Selection".

We encourage our authors to provide original source data, particularly uncropped/-processed electrophoretic blots and spreadsheets for the main figures of the manuscript. If you would like to add source data, we would welcome one PDF/Excel-file per figure for this information. These files will be linked as supplementary "Source Data" files.

It is Life Science Alliance policy that if requested, original data images must be made available to the editors. Failure to provide original images upon request will result in unavoidable delays in publication. Please ensure that you have access to all original data images prior to final submission.

If you are planning a press release on your work, please inform us immediately to allow informing our production team and scheduling a release date.

We welcome submissions of potential cover images for the issue of LSA in which your work would appear. If you have high quality images associated with this work, please feel free to email these, with a caption, to the journal office.

LSA encourages authors to provide a 30-60 second video where the study is briefly explained. These videos will be appear embedded with the manuscript online at Life Science Alliance, and on social media to promote the published paper and authors (for examples, see https://docs.google.com/document/d/1-UWCfbE4pGcDdcgzcmiuJl2XMBJnxKYeqRvLLrLSo8s/edit?usp=sharing). Corresponding or first-authors are welcome to submit the video. Please submit only one video per manuscript. The video can be emailed to contact@life-science-alliance.org

FINAL FILES:

To upload the final version of your manuscript, please log in to your account: https://lsa.msubmit.net/cgi-bin/main.plex
You will be guided to complete the submission of your revised manuscript and to fill in all necessary information. Please get in touch in case you do not know or remember your login name.

The following items are required for acceptance.

-- An editable version of the final text (.DOC or .DOCX) is needed for copyediting (no PDFs).

-- High-resolution figure, supplementary figure and video files uploaded as individual files: See our detailed guidelines for preparing your production-ready images, https://www.life-science-alliance.org/authors

-- Summary blurb (enter in submission system): A short text summarizing in a single sentence the study (max. 200 characters including spaces). This text is used in conjunction with the titles of papers, hence should be informative and complementary to the title. It should describe the context and significance of the findings for a general readership; it should be written in the present tense and refer to the work in the third person. Author names should not be mentioned.

The license to publish form must be signed before your manuscript can be sent to production. A link to the license to publish form will be available to the corresponding author only. Please take a moment to check your funder requirements.

Reviews, decision letters, and point-by-point responses associated with peer-review at Life Science Alliance will be published, alongside the manuscript. If you do want to opt out of having the reviewer reports and your point-by-point responses displayed, please let us know immediately.

Thank you for your attention to these final processing requirements. Please revise and format the manuscript and upload materials as soon as you are able.

Thank you for this interesting contribution to the literature. We look forward to publishing your paper in Life Science Alliance.

Sincerely,

Tim Fessenden
Scientific Editor
Life Science Alliance

Reviewer #1 (Comments to the Authors (Required)):

The revised manuscript remains primarily a technical feasibility study demonstrating that Xenium V1 (high sensitivity, low plex) and Xenium Prime (lower sensitivity, high plex) can be sequentially run on the same tissue section and computationally integrated. In response to reviewers, the authors added benchmarking data (localization comparisons, false-positive rates, probe cross-reactivity analyses), expanded discussions of scalability and compatibility, replaced the secretome analysis with a ligand-receptor analysis, and removed unconvincing clustering figure.
However, they did not provide additional biological validation, cell type/state annotation, multi-tissue validation, or a compelling demonstration that the dual chemistry approach yields biological discoveries unattainable by either assay alone.
My main concern ("what is the unique biological value that justifies the extra cost and complexity?") was acknowledged but only partially addressed through the new ligand-receptor analysis rather than through deeper biological validation.

However, if this is just seen as a proof of feasability, I agree that it can be published in this journal.

Reviewer #2 (Comments to the Authors (Required)):

The authors present a clever method to combine the high-sensitivity Xenium assay with high-breadth the Xenium assay, overcoming some of the trade-offs between targeted and discovery-based assays. This paper is very thorough and overall convinces me that the drawbacks in sensitivity associated with combining assays are relatively minimal, so users who cannot afford to trade off these elements should consider the approach. The authors addressed all of my concerns in the last review of this paper, so I am happy to recommend this for publication.

Reviewer #3 (Comments to the Authors (Required)):

The authors provided somewhat complete adjustments to the manuscript as per requests from the reviewers and clarified unclear parts of the manuscript and figures. My understanding is that the authors are currently unable to comprehensively dissect the biological relevance of the proposed approach due to parallel unpublished investigations, which in my opinion is the major limitation of the study.
Therefore, the present manuscript is only limited to describing the technical feasibility of the dual run approach. Despite it might be cost ineffective, it remains a useful piece of knowledge useful to the spatial transcriptomic community.

July 15, 2026

RE: Life Science Alliance Manuscript #LSA-2026-03690RR

Ms. Arianna L. Williams-Katek
Translational Genomics Research Institute
445 N 5th St
Phoenix, AZ 85004

Dear Dr. Williams-Katek,

Thank you for submitting your Methods entitled "Fishing with Two Lines: A Hybrid Approach to Spatial Transcriptomic Discovery". It is a pleasure to let you know that your manuscript is now accepted for publication in Life Science Alliance. Congratulations on this interesting work.

The final published version of your manuscript will be deposited by us to PubMed Central upon publication.

Your manuscript will now progress through copyediting and proofing. It is journal policy that authors provide original data upon request.

Your article will publish open access upon publication under a CC-BY license.

Reviews, decision letters, and point-by-point responses associated with peer-review at Life Science Alliance will be published, alongside the manuscript. If you do want to opt out of having the reviewer reports and your point-by-point responses displayed, please let us know immediately.

IMPORTANT: If you will be unreachable at any time, please provide us with the email address of an alternate author. Failure to respond to routine queries may lead to unavoidable delays in publication.

Scheduling details will be available from our production department. You will receive proofs shortly before the publication date. Only essential corrections can be made at the proof stage so if there are any minor final changes you wish to make to the manuscript, please let the journal office know now.

DISTRIBUTION OF MATERIALS:
Authors are required to distribute freely any materials used in experiments published in Life Science Alliance. Authors are encouraged to deposit materials used in their studies to the appropriate repositories for distribution to researchers.

You can contact the journal office with any questions, contact@life-science-alliance.org

Again, congratulations on a very nice paper. I hope you found the review process to be constructive and are pleased with how the manuscript was handled editorially. We look forward to future exciting submissions from your lab.

Sincerely,

Tim Fessenden
Scientific Editor
Life Science Alliance
